# Preventing post-surgical cardiac adhesions with a catechol-functionalized oxime hydrogel

**DOI:** 10.1038/s41467-021-24104-w

**Published:** 2021-06-18

**Authors:** Masaki Fujita, Gina M. Policastro, Austin Burdick, Hillary T. Lam, Jessica L. Ungerleider, Rebecca L. Braden, Diane Huang, Kent G. Osborn, Jeffrey H. Omens, Michael M. Madani, Karen L. Christman

**Affiliations:** 1 Department of Bioengineering, University of California, San Diego, San Diego, CA USA; 2 Sanford Consortium for Regenerative Medicine, University of California, San Diego, San Diego, CA USA; 3 Department of Medicine, University of California, San Diego, San Diego, CA USA; 4 Division of Comparative Pathology and Medicine, School of Medicine, University of California, San Diego, San Diego, CA USA; 5 Division of Cardiovascular and Thoracic Surgery, University of California, San Diego, San Diego, CA USA

**Keywords:** Biomedical materials, Biomedical engineering, Biomaterials

## Abstract

Post-surgical cardiac adhesions represent a significant problem during routine cardiothoracic procedures. This fibrous tissue can impair heart function and inhibit surgical access in reoperation procedures. Here, we propose a hydrogel barrier composed of oxime crosslinked poly(ethylene glycol) (PEG) with the inclusion of a catechol (Cat) group to improve retention on the heart for pericardial adhesion prevention. This three component system is comprised of aldehyde (Ald), aminooxy (AO), and Cat functionalized PEG mixed to form the final gel (Ald-AO-Cat). Ald-AO-Cat has favorable mechanical properties, degradation kinetics, and minimal swelling, as well as superior tissue retention compared to an initial Ald-AO gel formulation. We show that the material is cytocompatible, resists cell adhesion, and led to a reduction in the severity of adhesions in an in vivo rat model. We further show feasibility in a pilot porcine study. The Ald-AO-Cat hydrogel barrier may therefore serve as a promising solution for preventing post-surgical cardiac adhesions.

## Abstract (web summary)

Postsurgical adhesions are a problem during routine cardiothoracic procedures. Here, the authors report on a catechol functionalised hydrogel as an anti-adhesion material with improved retention on the heart which is biocompatible and biodegradable with minimal swelling, demonstrating application in vivo.

## Introduction

Depressed fibrinolytic activity resulting from surgical trauma to the epicardium during open-heart procedures can lead to fibrous adhesion formations between the epicardium and other tissues in the chest cavity^1–3^. Formation of these fibrous adhesions impedes heart function and severely complicates resternotomy by obstructing visibility and increasing the risk of mortality and morbidity during dissection^2,4–6^. For children born with congenital heart defects, who will experience multiple surgeries over their lifetime, and adults receiving valve replacements, mechanical circulatory support, and/or coronary artery bypass grafting, this problem is particularly relevant^2,7,8^. Reoperations for adult patients now constitute more than 20% of the annual caseload in cardiac surgery^9^.

A variety of materials have been studied for the reduction and prevention of surgical adhesions, such as Seprafilm (sodium hyaluronate/carboxymethylcellulose sheet) and CoSeal (polyethylene glycol (PEG) hydrogel)^10–15^. However, limited success has been demonstrated for preventing or reducing the severity of cardiac adhesions due to short retention time as a result of dynamic motion of the heart, short degradation times, and excessive swelling of the polymer leading to cardiac tamponade^10–12,14–18^. One product was approved in the US for preventing cardiac adhesions, REPEL-CV (polylactic acid/PEG sheet), although this failed to reduce adhesion dissection time^19^ and is no longer sold.

A promising method for ameliorating cardiac adhesion formation is to coat the epicardium with a fast-gelling polymer barrier to prevent susceptible tissue from adhering to the sternum or other organs in the chest cavity^4,12,17^. In situ polymerization/crosslinking of hydrogels after spraying has been tested for many adhesion prevention applications using a variety of different chemistries, including thiol-Michael addition reactions, “click” chemistry, amine-aldehyde imine-forming reactions, and photo-initiated radical crosslinking^20–26^. Designing these polymer coatings for the heart is, however, challenging because the polymer material must: (1) be easily applied; (2) rapidly gel on the tissue surface in an aqueous environment; (3) be retained on the epicardium for at least 2 weeks to overcome the initial inflammatory response post-surgery; (4) exhibit biocompatibility; and (5) have minimal swelling to prevent cardiac tamponade^4,13,14,27^. Ideally, the polymer could also be easily manually removed if a surgeon requires immediate re-access to the heart in case of emergency.

**Fig. 1:**
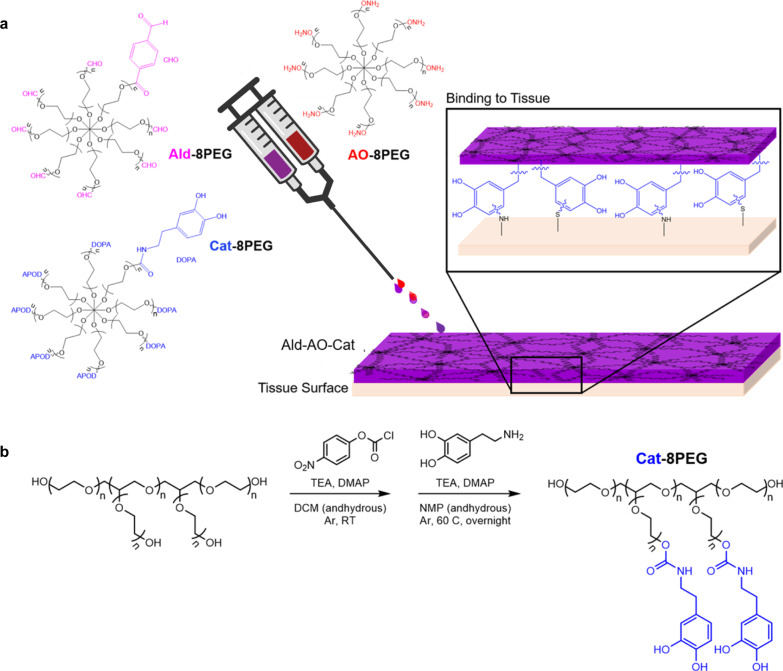
Fabrication of catechol-functionalized oxime hydrogel. **a** Three distinct 8-arm star PEG polymers were used to form the oxime hydrogel. The Ald-8PEG/Cat-8PEG solution is mixed with the AO-8PEG solution in a 1:1 ratio and sprayed onto the tissue surface using a FibriJet gas-assisted applicator head from Nordson Micromedics. Oxime bonds rapidly form between Ald-8PEG and AO-8PEG. The catechol forms covalent bonds with the primary amines present on the tissue surface to promote hydrogel-tissue attachment. **b** Synthesis of Cat-8PEG required a two-step process by activating PEG–OH with NPC and then deprotecting with dopamine hydrochloride. Ald-8PEG and AO-8PEG were synthesized as previously reported^46^. Poly(ethylene glycol) PEG, aldehyde Ald, catechol Cat, aminooxy AO, triethylamine TEA, 4-(dimethylamino) pyridine DMAP, dichloromethane DCM, N-methyl-2-pyrrolidone NMP, 4-nitrophenyl chloroformate NPC.

Our earlier studies identified an oxime-crosslinked star PEG-based injectable hydrogel consisting of aldehyde-terminated PEG and aminooxy terminated PEG (Ald–AO) as an approach to potentially prevent post-operative pericardial adhesions^28^. PEG-based hydrogel systems are resistant to protein and cell adhesion, and oxime bonds form rapidly and have excellent hydrolytic stability^28–31^. Unlike other hydrogel systems with high swelling ratios (~400%) that have been studied for cardiac anti-adhesion applications, this Ald–AO PEG-oxime hydrogel rapidly forms gels with high crosslink density, allowing for a reduced swelling ratio, which should minimize the risk of cardiac tamponade when translated in vivo^14,32^. Despite the ability of aldehydes to bind to amines on the epicardial surface, preliminary in vivo studies in a rat model showed that retention of the material on the heart was not optimal, which led to inconsistent results with adhesion prevention (unpublished). Herein, we developed a three-component PEG-based, injectable oxime hydrogel system (Ald–AO–Cat), utilizing the mussel-inspired catechol (Cat) found on dopamine (DA) for prolonged retention time on cardiac tissue and prevention of adhesion formation (Fig. 1a). Cat compounds, such as DA, participate in strong covalent and non-covalent interactions with both organic and inorganic substrates. Particularly, mussel adhesive proteins are enriched in 3,4-dihydroxy-phenylalanine (DOPA), which is attributed to its ability to bind strongly to surfaces under water^33–35^. When exposed to air, catecholic compounds are oxidized to quinones which further react with nucleophiles, such as amines^36–38^. Cat chemistry has been studied for many biomedical applications, particularly as tissue adhesive materials, with much success^39–44^, which motivated our use of it to prolong polymer retention on the heart. Interestingly, in one study using a Cat-functionalized PEG hydrogel for islet transplantation in a murine model, researchers made note of the complete absence of non-specific adhesions to the implant, which was attributed to the high PEG content of the hydrogel^45^. However, to the best of our knowledge, a Cat-conjugated hydrogel has never been utilized in a material designed to be applied to and prolong retention on an organ to prevent post-surgical adhesions.

Herein, we tested the use of the Ald–AO–Cat gel for specifically preventing post-surgical cardiac adhesions through studying its mechanical and biological properties via parallel-plate rheometry, tissue retention assays, and cellular adhesion over time. Additionally, this study demonstrates the cytocompatibility of the hydrogel through metabolic assays performed after direct contact with an in vitro cell layer. Lastly, the Ald–AO–Cat system was implanted into small and large animal cardiac adhesion models to investigate its performance on the surface of the heart. We have demonstrated the use of this hydrogel barrier system in reducing the severity and preventing cardiac adhesions in a rat model of post-surgical cardiac adhesions as well as in a pilot porcine study, indicating its promising clinical application in cardiothoracic procedures.

## Results

### Synthesis and characterization of polymers

This oxime-hydrogel system required the synthesis of three distinct 8-arm PEG polymers: aldehyde-functionalized (Ald-8PEG), aminooxy-functionalized (AO-8PEG), and Cat-functionalized (Cat-8PEG). Ald-8PEG and AO-8PEG were synthesized as previously reported^46^. Cat-8PEG was synthesized in two steps by activation of 8-arm PEG-OH with 4-nitrophenyl chloroformate (NPC) followed by deprotection with DA hydrochloride (Fig. 1b). ^1^H NMR (Supplementary Figs. 1–3) was used to confirm the successful synthesis and the functionalization ratio of each PEG component. Cat-functionalization was further verified using UV–Vis (Supplementary Fig. 4) because of the hygroscopic nature of PEG and the overlap of H_2_O and PEG proton peaks in the ^1^H NMR. The average number of functionalized arms was calculated and is reported in Table 1. Functionalization ratios of polymers ranged from 74% to 97%, which can be equated to the successful functionalization of 5.9–7.8 arms of the PEG–OH polymer for each PEG polymer.

**Table 1:** Polymer functionalization.

Polymer	Functionalization^a^ (%)	Functionalization^b^ (%)	Number of arms functionalized
Ald-8PEG	97	–	7.8
AO-8PEG	83	–	6.6
Cat-8PEG	74	81	5.9^a^ (6.5)^b^

^a^Calculated from ^1^H NMR peak integrations.

^b^Calculated from UV–Vis.

### Formation and characterization of hydrogels

All hydrogels were formed with a fixed 1:1 ratio of Ald-8PEG:AO-8PEG because excess aldehyde groups significantly affected the metabolic activity of 3T3 fibroblasts with an elution extract as previously reported^46^. All gel formulations are listed in Table 2.

**Table 2:** Hydrogel formulations.

Hydrogel	Mass concentration in final gel (mg/mL)			Cat-8PEG in hydrogel (wt%)	Gelation time (s)
	Ald-8PEG	AO-8PEG	Cat-8PEG		
Ald–AO	100	100	–	0	<3
Ald–AO–Cat	100	100	50	20.0	<3
	**100**	**100**	**75**	**27.8**	**<3**
	100	100	100	33.3	<3
	100	100	125	38.5	<3
	100	100	300	60.0	<3

Final formulation in bold.

**Fig. 2:**
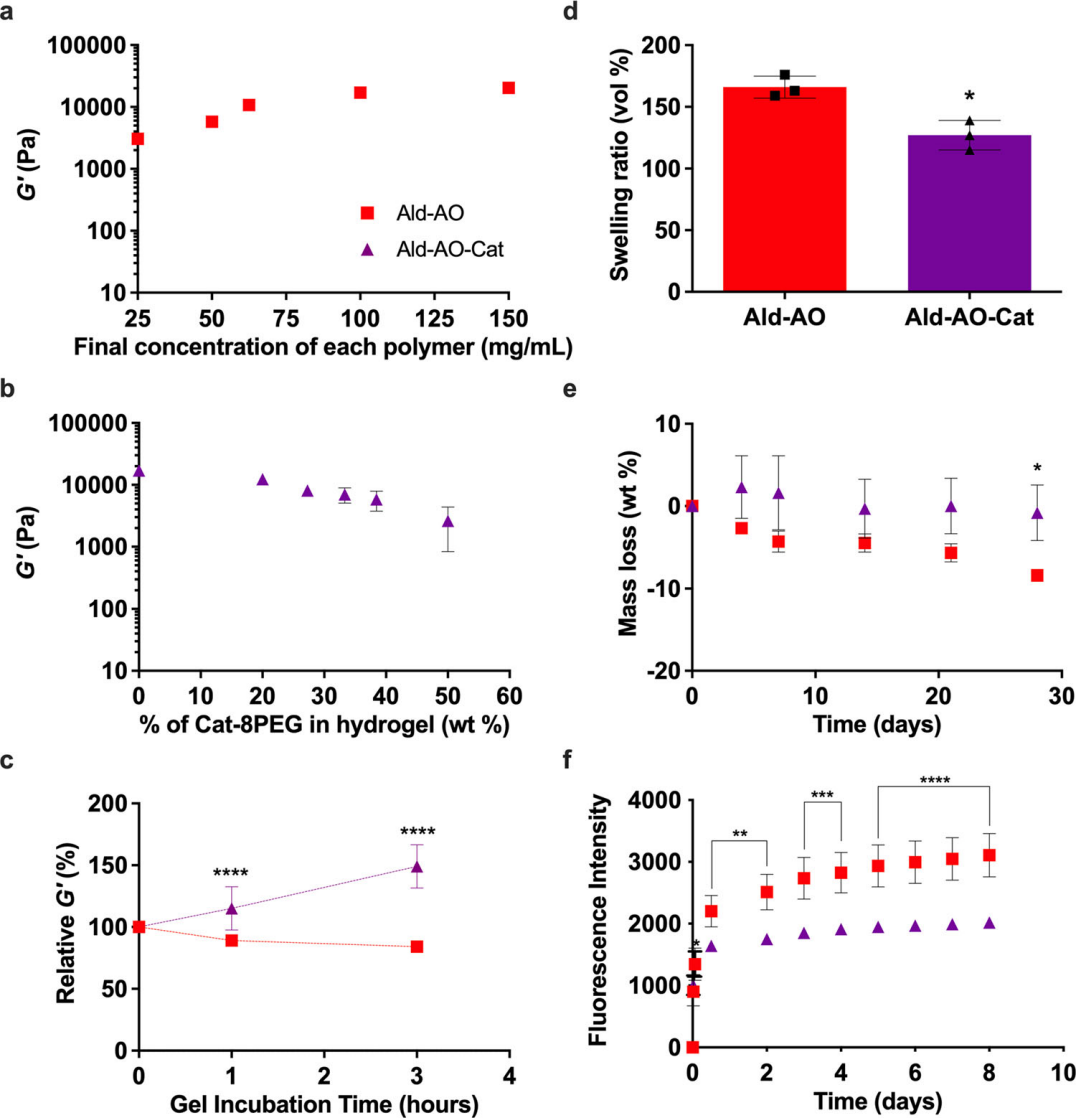
Characterization of rapidly gelling oxime hydrogels. **a** Storage moduli (*G*′) were measured as a function of final polymer mass concentration in Ald–AO gels using a frequency sweep from 10^−2^ to 10^2^ Hz at 37°C; *G*′ at 1Hz is shown. **b** Ald–AO–Cat gels with varying Cat-8PEG content were gelled and *G*′ was measured under the same conditions. Increasing Cat-8PEG content of the gels resulted in decreasing *G*′. **c** *G*′ for Ald–AO–Cat and Ald–AO were measured up to 3h post gelation and relative *G*′ was calculated. Over the 3h incubation period, *G*′ significantly increased for Ald–AO–Cat indicating the formation of secondary crosslinks between Cat and AO. **d** Low swelling ratios were observed for both Ald–AO–Cat and Ald–AO gels, with significantly lower swelling ratio observed for Ald–AO–Cat. **e** In vitro degradation of the oxime hydrogels revealed significantly greater mass loss after 28 days for Ald–AO gels compared to Ald–AO–Cat gels. **f** Ex vivo retention of Ald-AO-Cat shows significantly greater retention on the tissue surface compared to Ald-AO over 8 days. Note that some error bars are too small to be visible in **a**–**c**, **e**, **f**. Mean ± SD and analyzed with a two-way ANOVA with a Tukey’s post-hoc test in **c**, **e**, **f** and an unpaired two-sided *t*-test in **d** (*p < 0.05, **p < 0.005, ***p < 0.001, ****p < 0.0001 between hydrogels). *n* = 21 individual frequencies were used to determine the means in **a**–**c**. Measurements in **a** were repeatedly independently with similar results for 100mg/mL (*n* = 3) and 62.5mg/mL (*n* = 3), and measurements in **b** were repeatedly independently with similar results for 0% (*n* = 3) and all other concentrations (*n* = 2). *n* = 3 independent gel samples for each condition in **d**–**f**. Source data are provided as a Source Data file.

Storage modulus (*G*′) for Ald–AO gels was studied as a function of polymer concentration (Fig. 2a). *G*′ values ranged from 3.0 ± 0.3 to 20.1 ± 1.2 kPa for 25 and 150 mg/mL of total polymer, respectively. For 100 mg/mL, *G*′ was 17.0 ± 0.6 kPa; this polymer concentration was chosen for the Ald–AO gel as well as for further testing with the incorporation of Cat-8PEG. The ratio of Cat-8PEG was varied for Ald–AO–Cat gel formation to find the optimal hydrogel parameters. All formulations gelled rapidly (<3 s) regardless of Cat-8PEG content (Table 2). Increased Cat-8PEG content in Ald–AO–Cat gels resulted in a decreased *G*′ (Fig. 2b), ranging from 17.0 ± 0.6 to 2.6 ± 1.8 kPa for 0 wt% Cat-8PEG and 50 wt% Cat-8PEG incorporation, respectively. The Cat-oxime hydrogel consisting of 100 mg/mL of Ald-8PEG and AO-8PEG and 75 mg/mL (27.8%) of Cat-8PEG (*G*′ = 8.1 ± 1.0 kPa) was evaluated in subsequent studies. Relative *G*′ was calculated at 1 and 3 h, compared to *G*′ at the initial gel formation (Fig. 2c). *G*′ significantly increases from 0 to 3 h for Ald–AO–Cat, compared to Ald–AO, which ultimately shows no change in *G*′. At 3 h post gelation, *G*′ for Ald–AO–Cat increased 150 ± 20%. A representative frequency sweep of *G*′ and *G*″ exhibits the viscoelastic behavior of the crosslinked gels (Supplementary Fig. 5). Rheometry was also used to calculate the individual polymer solution viscosities, which were 4.2 × 10^−2^ and 1.0 × 10^−2^ Pa s for Ald/Cat-8PEG and AO-8PEG solutions, respectively (Supplementary Fig. 6).

The Ald–AO–Cat and Ald–AO gels exhibited 127 ± 12% and 166 ± 9% swelling, respectively, in PBS (pH 7.4) after reaching swelling equilibrium in 24 h (Fig. 2d). Degradation of Ald–AO–Cat and Ald–AO gels was tested in vitro for 28 days in PBS (pH 7.4). A mass loss of 8.4 ± 0.4 wt% was observed for Ald–AO gel, which was significantly greater than the 0.8 ± 3.4 wt% mass loss observed for Ald–AO–Cat after 28 days (Fig. 2e).

Retention of the oxime hydrogel systems was tested in PBS (pH 7.4) on ex vivo porcine cardiac biopsy punches using fluorescently labeled Ald-8PEG and AO-8PEG (Fig. 2f). For both the Ald–AO and Ald–AO–Cat gels, a burst release of the polymer was observed within the first 12 h. However, after 2 days, significantly less fluorescently labeled polymer was released from Ald–AO–Cat gels, compared to the Ald–AO gel. This pattern was observed for 8 days. Additionally, the retention of Cat-8PEG was observed in a time-dependent release study over 7 days. Similar to the ex vivo retention data, a burst release of Cat-8PEG was seen in the day 1 supernatant, with decreasing concentration of Cat-8PEG released over time (Supplementary Fig. 7a). The total amount of Cat-8PEG released and the remaining Cat-8PEG in the gel after 7 days of incubation was compared to the total Cat-8PEG content in the gel formulation at the time of gelation. About 37% of the Cat-8PEG was not accounted for using this method, which is attributed to the incomplete digestion of the Cat-8PEG gel at day 7, resulting in additional Cat-8PEG sticking in the gel pellet and not being detected with the assay reading (Supplementary Fig. 7b, c).

### In vitro cell studies

**Fig. 3:**
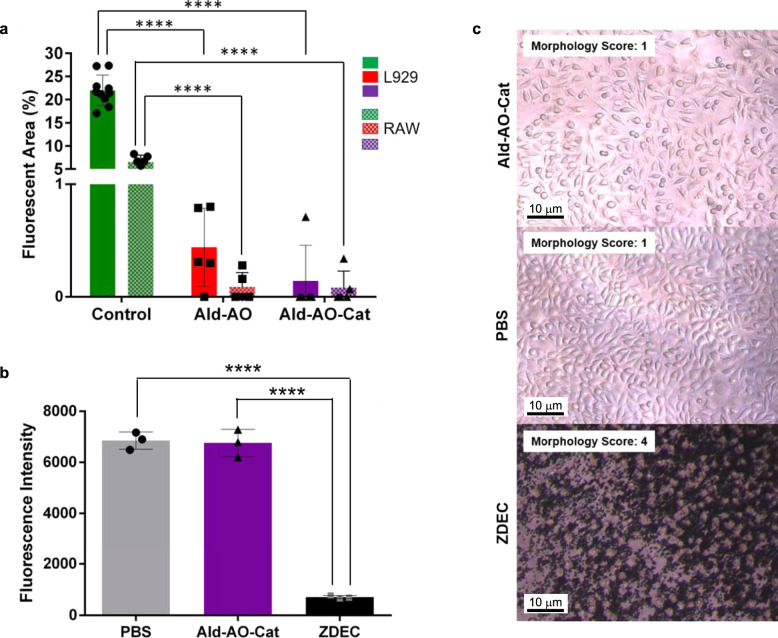
In vitro cell adhesion and cytocompatibility testing of Ald–AO–Cat. **a** Inflammatory cell adhesion was studied on oxime hydrogels and tissue culture plastic as a control. L929 fibroblasts and RAW macrophages were seeded on each surface and imaged after 24 h of incubation for cell adhesion, as indicated by the fluorescent area. <1% fluorescent area was observed for both gels for L929 and RAW cells. Conversely, 22% and 6.6% fluorescence area were observed for L929 and RAW in control wells, indicating oxime hydrogels resist inflammatory cell adhesion. *n* = 10, 5, and 5 independent wells with L929 cells for control, Ald–AO, and Ald–AO–Cat, respectively. *n* = 6, 5, and 5 independent wells with RAW cells for control, Ald–AO, and Ald–AO–Cat, respectively. **b** Cytocompatibility of Ald–AO–Cat gels was assessed after 24 h of incubation in direct contact with the L929 fibroblast cell monolayer. Phosphate-buffered saline (PBS) and zinc diethyldithiocarbamate (ZDEC) was doped into the media for positive and negative controls, respectively. Ald–AO–Cat did not significantly affect metabolic activity compared to the positive control. *n* = 3 independent samples for each condition. **c** Representative brightfield images of the fibroblasts in each condition (*n* = 9 independent wells per group) reveal that no effect on cell morphology and spreading was observed for Ald–AO–Cat compared to the positive control. A cell morphology score was assigned to each brightfield image for quantification of cell morphology. Morphology scores suggest that fibroblasts are well spread and that no apparent cell lysis occurs when in direct contact with Ald–AO–Cat. Data are reported as mean ± SD and analyzed with a one-way ANOVA with a Tukey’s posthoc test (****p < 0.0001). Source data are provided as a Source Data file.

L929 fibroblast and RAW macrophage adhesion to Ald–AO–Cat and Ald–AO gels were compared to control tissue culture (TC) plastic adhesion (Fig. 3a). Cells were seeded on each surface and fluorescent images of prelabeled cells were taken to determine percent surface coverage after 24 h incubation. <1% fluorescent area was observed for both cell lines on hydrogel surfaces, while 6.5 ± 1.5% and 22.0 ± 3.3% fluorescent area was observed for RAW macrophages and L929 fibroblasts, respectively, on TC plastic controls.

Cytocompatibility of the Ald–AO–Cat gels was tested with a direct contact assay by forming the Ald–AO–Cat gel directly on an L929 fibroblast monolayer. Cells were incubated for 24 h and cytocompatibility was assessed via metabolic activity (Fig. 3b) and imaging (Fig. 3c) against a positive and a negative control. After 24 h incubation, alamar blue readings indicate that there was no effect on metabolic activity in the presence of Ald–AO–Cat. The resulting fluorescent intensities showed no significant difference from PBS controls (6760 ± 538 compared to 6855 ± 340, respectively). Brightfield images were obtained for assessing cell morphology for each treatment group (Fig. 3c). Images were blinded from the grader and morphology scores ranging from 1 to 4 were assigned. Morphology scores are defined in “Methods” section. When treated with Ald–AO–Cat (*n* = 9) and PBS (*n* = 9), cells consistently received a score of 1 (≤20% of cells appear rounded with minimal lysing), while all ZDEC control wells (*n* = 9) were given scores of 4 (all cells appear rounded and lysed). Both the alamar blue and morphology scores indicate the cytocompatibility of Ald–AO–Cat.

### In vivo rat cardiac adhesion model

**Fig. 4:**
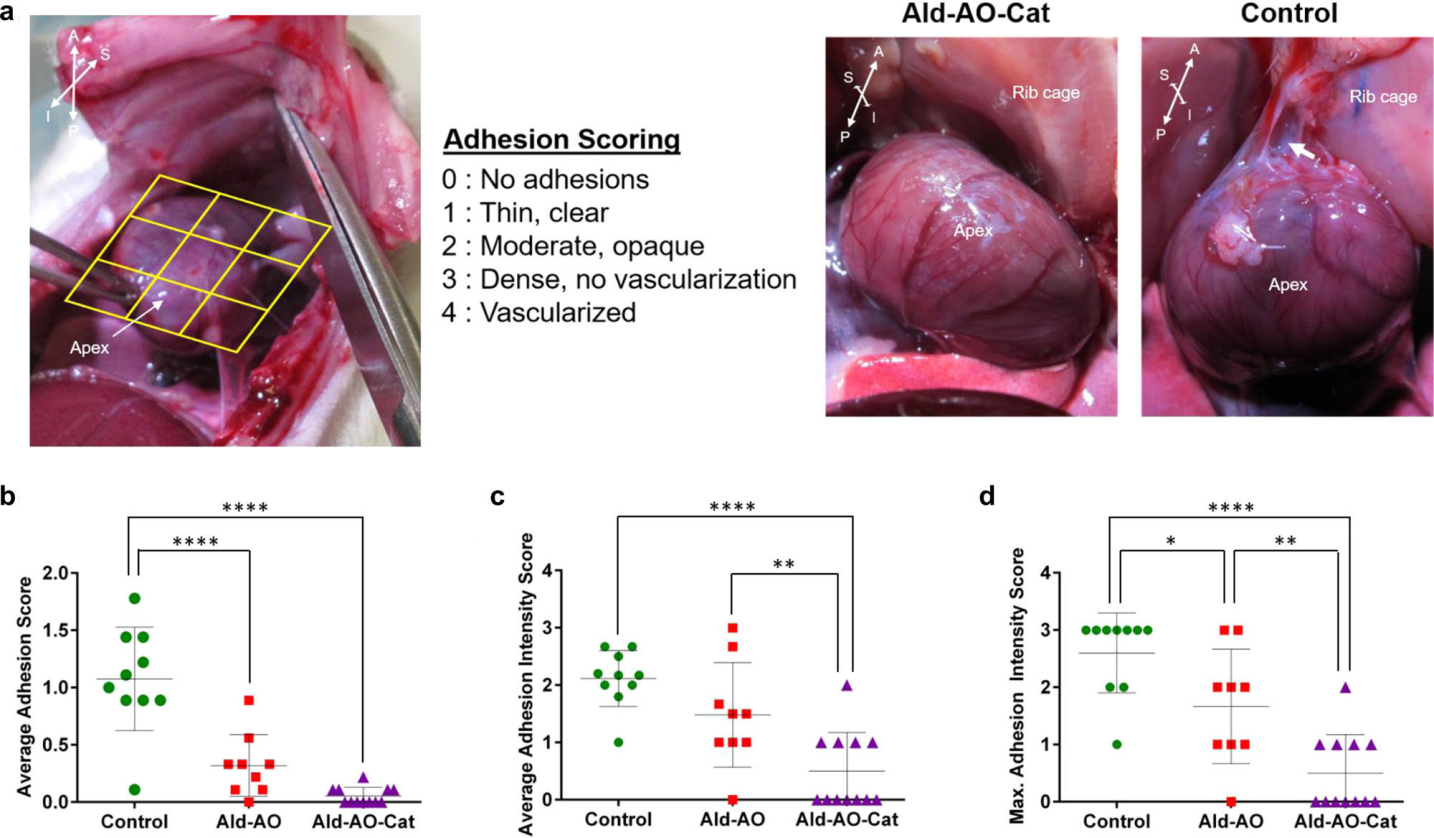
Reduced adhesion formation 2 weeks after application in rat surgical model. Adhesion formation was  assessed for administration of Ald–AO–Cat, Ald–AO, and the untreated control after 2 weeks. **a** Adhesion scores were assigned to 9 discrete segments of the exposed heart surface when the chest was re-entered during euthanasia and harvest. Representative images of adhesions upon re-entry for Ald–AO–Cat and control groups are shown. Anatomical directions are shown to provide a point of reference: A—anterior, P—posterior, S—superior, I—inferior. **b** The individual scores for the 9 segments were averaged to obtain the adhesion score for each heart. At 2 weeks there was a significant reduction in adhesion score for Ald–AO–Cat and Ald–AO compared to the untreated group. **c** The average intensity score was calculated over regions of adhesion formation. At 2 weeks, Ald–AO–Cat show significantly reduced adhesion intensity compared to Ald–AO and the untreated control. **d** The maximum adhesion intensity score was also reported for each animal. Significantly higher maximum intensity scores were assigned to adhesions in rats treated with Ald–AO and the untreated group, compared to Ald–AO–Cat. Together these data suggest reduced adhesion formation and intensity when Ald–AO–Cat is applied following abrasion. Data are reported as mean ± SD and analyzed with a one-way ANOVA with a Tukey’s posthoc test (*p < 0.05, **p < 0.005, ****p < 0.0001) *n* = 12, 9, and 10 independent rats for Ald–AO–Cat, Ald–AO, and Control groups, respectively. Source data for **b**–**d** are provided as a Source Data file.

A rat cardiac adhesion model was used to test the ability of the Ald–AO–Cat gel to prevent cardiac adhesions compared to Ald–AO gel and untreated rats. A gross assessment of cardiac adhesion formation was performed when the chest was re-entered. Before dissecting the adhesions to harvest the heart, images were taken and given to blinded graders for assigning adhesion scores. The heart was divided into 9 segments, and each segment was given an overall adhesion score based on the presence and severity of adhesions from 0 to 4 (Fig. 4a). The average score over these 9 segments was reported as the average adhesion score. The average adhesion intensity was calculated over all regions that showed adhesion formation. The maximum adhesion intensity score was also reported for each animal. This is a common method used for adhesion scoring in various in vivo models^47–49^. Representative images of the observed adhesions in control and Ald–AO–Cat groups are shown in Fig. 4a.

At 2 weeks, there was a significant reduction in average adhesion score when Ald–AO–Cat and Ald–AO were applied, compared to the untreated control (Fig. 4b). All of the treatment groups consistently received low adhesion scores;Ald–AO–Cat (0.1 ± 0.1) and Ald–AO (0.3 ± 0.3) groups were significantly lower compared to the untreated (1.1 ± 0.5) control at 2 weeks. The average intensity scores of the adhesions showed similar results, with a significantly reduced adhesion intensity score when Ald–AO–Cat (0.5 ± 0.7) was applied, compare to Ald–AO (1.5 ± 0.9) and the untreated control (2.1 ± 0.5). There was no difference in average adhesion intensity score reported for Ald–AO and untreated groups. Maximum adhesion intensity score was the final parameter used to grade adhesion formation and hydrogel efficacy. Compared to Ald–AO (1.7 ± 1.0) and untreated groups (2.6 ± 0.7), Ald–AO–Cat (0.5 ± 0.7) application resulted in significantly lower maximum adhesion intensity.

**Fig. 5:**
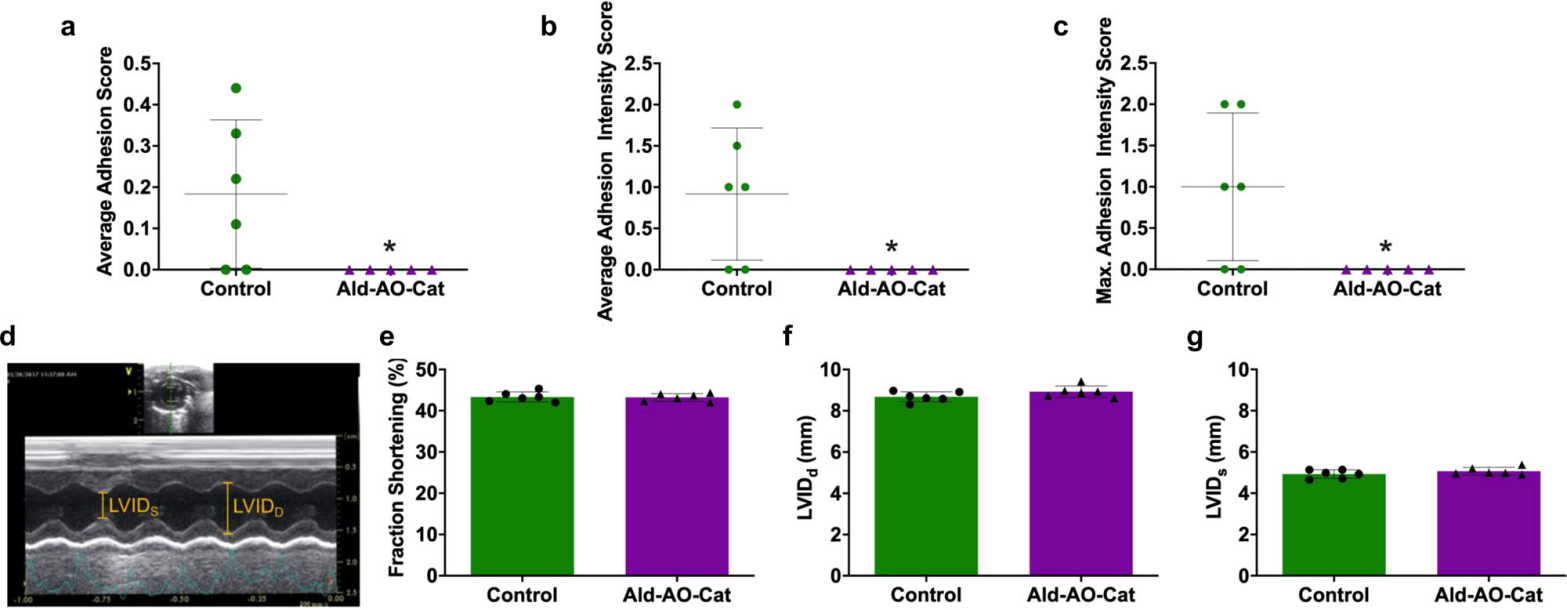
Reduced adhesion formation and maintained cardiac function 4 weeks after application in rat surgical model. Adhesion formation and heart function were assessed for administration of untreated control and Ald–AO–Cat. **a**–**c** At 4 weeks, all parameters for comparing adhesion formation and intensity showed significantly lower values for Ald–AO–Cat when compared to the untreated control. It is also worth noting that all Ald–AO–Cat-treated animals received an average adhesion and intensity score of 0, indicating the complete reduction of adhesion formation. **d**–**g** Cardiac function was assessed 3 ± 1 days post material application using M-mode echocardiography. Fraction shortening was calculated from measured left ventricle internal diameter systole (LVID_s_) and left ventricle internal diameter diastole (LVID_D_), and regardless of treatment, animals showed identical cardiac function, indicating no adverse effects from the application of Ald-AO-Cat gels. Data are reported as mean ± SD and analyzed with an unpaired two-sided Mann–Whitney *t*-test (*p < 0.05). *n* = 5 and 6 independent animals for Ald–AO–Cat and Control groups, respectively, in **a**–**g**. Source data for **a**–**c** and **e**–**g** are provided as a Source Data file.

Adhesion formation and cardiac function were assessed in a second in vivo study, comparing untreated rats to Ald–AO–Cat, since it was shown to be superior to the Ald–AO gel in the first study. At 4 weeks, a reduction in adhesion formation and severity was observed (Fig. 5). All of the rats treated with Ald–AO–Cat showed no signs of adhesion formation and received an overall adhesion score of 0, which was significantly lower than the untreated group (0.2 ± 0.2) (Fig. 5a). Consistent with 2-week results, the average adhesion intensity score (Fig. 5b) and maximum adhesion intensity score (Fig. 5c) for Ald–AO–Cat (scores of 0 for both measurements) were also significantly lower than the untreated group (average adhesion intensity 0.9 ± 0.8, maximum adhesion intensity 1.0 ± 0.9).

To ensure the hydrogels did not impede cardiac function, M-mode echocardiography was conducted 3 ± 1 days post material application (Fig. 5d–g). Three days were chosen to allow for full swelling in vivo to ensure that cardiac tamponade would not occur. When Ald–AO–Cat was applied there was no difference in end-diastolic left ventricular internal diameter (LVID_D_), end-systolic left ventricular internal diameter (LVID_S_), or fractional shortening (FS) compared to the untreated group, indicating normal cardiac function following gel application.

**Fig. 6:**
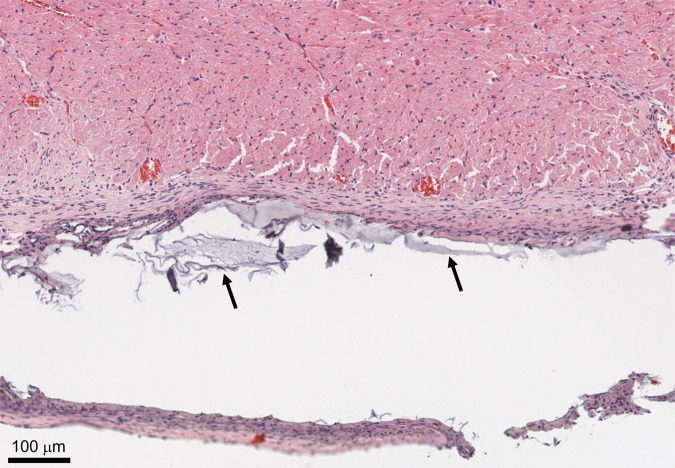
Histology of Ald–AO–Cat in a rat model. H&E staining was performed for Ald–AO–Cat tissue samples (*n* = 6 independent animals) to verify the biocompatibility of the oxime hydrogel systems. At 4 weeks, thin capsule formation around some remaining gel (black arrows) was visible, however, there was no indication of chronic toxicity as a result of application or degradation of the hydrogel system. Furthermore, no cell infiltration into the Ald–AO–Cat gel coincides with our expectations, due to the non-cell adhesive nature of PEG polymers.

Histological assessment was performed by a trained histopathologist on hearts that were harvested at 4 weeks. Hematoxylin and eosin (H&E) staining revealed the presence of some regions with Ald–AO–Cat hydrogel on the epicardium at 4 weeks, indicating that the material was not fully degraded. Minimal macrophage infiltration was visible at high magnification. In areas with patches of remaining hydrogel, there was evidence of some thin encapsulation (Fig. 6), however, there was no indication of lymphocyte and neutrophil infiltration, suggesting that no chronic inflammation was occurring and there was a resolved wound healing response.

### Dual-delivery device design and testing

**Fig. 7:**
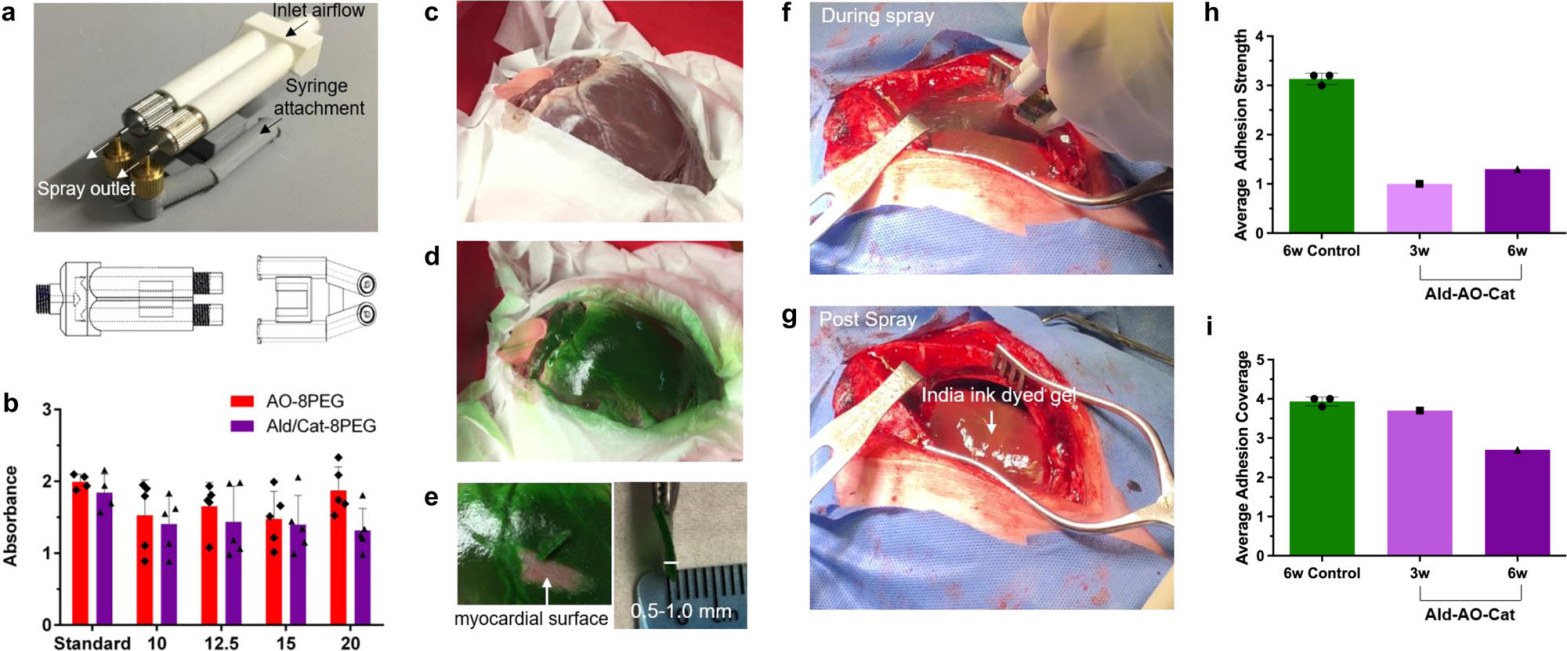
Spray device design, testing, and application in a pilot porcine study. **a** A specialized device was developed to deliver each component together in a 1:1 ratio in the form of an atomized spray for application in a large animal model. This device has two separate parts, one of which allows the flow of air from an air compressor through two separate pathways, while a second piece supports the addition of two syringes containing sterile solutions of the component polymers injected into the path of air through two pathways. **b** The mixing ratio of the sprayed solutions at various working distances was tested using mock Ald–Cat-8PEG and AO-8PEG solutions dyed blue and yellow, respectively. The standard was prepared by pipetting equal volumes of both solutions as a control (*n* = 4 independent samples). Equal mixing of both solutions was observed at all spray distances (*n* = 5 independent samples for each working distance and polymer). **c** The dual spray device was tested on an ex vivo porcine heart to determine an effective working distance and the amount of spray necessary for complete coverage of the anterior surface with a gel ~0.5–1.0 mm thick. The heart was set in a shallow insulated ice bucket surrounded by Kim wipes to simulate the exposure of the epicardium during a sternotomy. **d** The anterior surface of the heart was sprayed from a working distance of ~10–12 cm with a total of 8 mL (4 mL AO-8PEG (dyed blue) and 4 mL Ald–Cat-8EPG (dyed yellow)) of hydrogel solution. Equal mixing was visually determined by the green gel that formed on the surface of the heart. Minimal overspray is seen on the Kim wipes surrounding the heart surface, indicating effective targeting of the spray. **e** For clinical translation, in the case of an emergency, it would be advantageous that the gel could be manually removed by the surgeon. The gel thickness was measured after removal, showing the targeted 0.5–1.0 mm gel thickness has been obtained with the 8 mL of solution. **f**, **g** The Ald–AO–Cat gel applied using the dual spray device was tested in a pilot porcine study against a non-treated control. The solutions were prepared with a 1:100 dilution of India Ink for visualization of the gel once sprayed. **h** and **i** 3 and 6 weeks post-sternotomy and abrasion, resternotomy and adhesion scoring was performed based on the defined adhesion scoring system (Table 3). Assessment of adhesions revealed a decrease in adhesion strength in Ald–AO–Cat-treated animals (*n* = 1 pig at 3 weeks, *n* = 1 pig at 6 weeks), compared to controls (*n* = 3 pigs at 6 weeks). The treated animals required only simple blunt dissection of the adhesions to access the anterior surface of the heart, while the control required sharp dissection, resulting in tissue damage with adhesion removal. Average adhesion coverage was similar for all animals with a trending decrease in adhesion coverage at 6 weeks for the Ald–AO–Cat-treated animal. This observation agrees with clinical observations of reduced cardiac adhesion coverage at longer time points post sternotomy and surgical trauma. Data are reported as mean ± SD. Source data for **b**, **h**, **i** are provided as a Source Data file.

For delivery of our hydrogel system in a large animal model, an air-assisted dual delivery device was designed (Fig. 7a). The device design allowed for optimal 1:1 delivery ratio of both Ald–Cat-8PEG and AO-8PEG solutions, over the entire anterior surface of the heart as confirmed by a colorimetric spray test (Fig. 7b). At all distances from the sprayed surface, the mixing ratio showed no significant difference compared to the standard 1:1 mixed solution. Spraying of the device with yellow and blue-dyed PEG solutions onto epicardial tissue ex vivo resulted in a 0.5–1.0 mm-thick homogenous gel on the tissue surface, which could be manually removed if necessary, with minimal overspray onto “surrounding tissue” when spraying from a working distance of 12–17 cm with 10 psi air pressure (Fig. 7c–e).

### In vivo porcine cardiac adhesion model pilot study

A porcine cardiac adhesions model was used to test the ability of Ald–AO–Cat to reduce the coverage and severity of cardiac adhesions compared to non-treated controls in a pilot investigation. Following a full sternotomy, the heart was abraded with gauze, and either treated with Ald–AO–Cat or left untreated (Fig. 7f, g). The heart surface was left exposed to air and the chest cavity was closed. Resternotomy and adhesion assessment was performed 3 weeks (*n* = 1) and 6 weeks (*n* = 1) post-gel application, and 6 weeks post-abrasion for all control animals (*n* = 3) based on the scoring system defined in Table 3. The control pigs received an average adhesion coverage score of 3.9, while 3 and 6 weeks Ald–AO–Cat-treated pigs received scores of 3.7 and 2.6, respectively (Fig. 7h). More importantly, despite the formation of adhesions in all animals, the control pigs scored higher in adhesion strength (3.1) compared to 3-week and 6-week Ald–AO–Cat pigs (1.0 and 1.4, respectively) (Fig. 7i). This semi-quantitative assessment of adhesion strength shows that most adhesions in the control pigs required sharp dissection and resulted in tissue damage when removed, while gel-treated pigs reduced the severity of adhesions, which showed minimal fibrosis and required simple blunt dissection only. Representative images prior to dissection are shown in Supplementary Fig. 8.

**Table 3:** Pilot porcine adhesion scoring criteria.

Score	Adhesion coverage (%)	Adhesion strength
0	0	No adhesions
1	1–25	Weak adhesions, remove with little force
2	26–50	Moderately strong adhesions, blunt dissection required
3	51–75	Strong adhesions, requires sharp dissection
4	76–100	Very strong adhesions, requires sharp dissection resulting in additional tissue damage

## Discussion

In this study, oxime-crosslinked PEG hydrogels were synthesized and characterized for use as a cardiac anti-adhesion barrier. The oxime bond was chosen because previous work has demonstrated its rapid gel formation, chemo-specificity, aqueous stability, and biocompatibility^28,29,46,50^. The addition of Cat-8PEG was reported in this study, as the third component of this second generation oxime-hydrogel system for cardiac anti-adhesion applications. Inspired by nature, this polymer utilizes the adhesive properties of the Cat group in DA, a compound abundantly found in mussel proteins, which enables adhesion to a variety of surfaces in wet conditions^51^. The hydroxyl groups of Cat-8PEG react with free amines^38^, which exist on the epicardium, leading to an observed increased retention time on the tissue (Fig. 2f).

All hydrogels were formed with a 1:1 ratio of Ald-8PEG:AO-8PEG, to ensure that unreacted aldehyde groups would not interfere with cell metabolic activity, as previously reported^46^. While maintaining a fixed 1:1 ratio, the polymer concentration was varied to find the optimal formulation for Ald-AO gels. *G*′ is tunable with varying polymer concentration, with an observed increase in *G*′ with increasing polymer concentration. All polymer concentrations exhibited *G*′ within the range for cardiac muscle (~10–100 kPa), which is crucial for mimicking the elasticity of the native cardiac tissue^52–55^. Gelation time (<3 s) and solution viscosities were taken into consideration when deciding the 100 mg/mL concentration would be the primary formulation for the Ald–AO gel. Tunable *G*′ was also observed when the concentration of Cat-8PEG was varied for the Ald–AO–Cat formulation. A trending decrease in *G*′ was observed with an increase in Cat-8PEG wt%. The 27.8 wt% Ald–AO–Cat formulation was considered optimal because the solution viscosities did not complicate the delivery for gel formation, rapid gelation was achieved, and it resulted in a storage modulus that should not interfere with cardiac function. Overall, we wanted to use the highest wt% Cat-8PEG possible to ensure longer retention times on the tissue surface when applied in vivo without compromising ease of delivery through viscous solutions. Relative *G*′ measurements of Ald–AO–Cat resulted in an increase in *G*′ as incubation time increased, suggesting that physical entrapment of Cat-8PEG increases the storage modulus because of limited chain movement. This phenomenon was not observed for Ald–AO.

Swelling and degradation were used to characterize Ald–AO and Ald–AO–Cat gel formulations. At physiological pH in PBS, there was minimal swelling (<200%) for both hydrogel systems. In clinical trials, the excess swelling (400%) of CoSeal^®^ was reported to be the cause of cardiac tamponade in several patients^13,14^. Degradation of both oxime hydrogel systems in PBS pH 7.4 over the course of 28 days revealed significantly greater degradation of the Ald–AO gel compared to Ald–AO–Cat. Slow degradation of Ald–AO–Cat revealed <1% degradation over 28 days in physiological conditions.

Retention times of Ald–AO–Cat and Ald–AO were evaluated on ex vivo porcine biopsy punches over the course of 8 days. The goal was to develop a cardiac anti-adhesion barrier that would be retained on the native tissue for at least 2 weeks, to overcome the initial inflammatory response post-surgery^56^. In preliminary unpublished work with the Ald–AO gel in vivo, we found this formulation had inadequate retention on the heart. In this study we sought to develop a material with better retention, which we hypothesized would form a more robust anti-adhesion barrier since several barrier materials have failed as cardiac anti-adhesion materials because of short retention and short degradation times^10–12,15–17^. We demonstrated that 27 wt% addition of Cat-8PEG in the PEG-oxime hydrogel system allows for greater retention on porcine cardiac tissue compared to Ald–AO. With this Ald–AO–Cat composition, the Ald-8PEG and Cat-8PEG are both available for binding to excess amines on the tissue surface for longer retention. Retention data also suggests that Cat-8PEG is retained in the gel via physical entrapment, which can help explain the longer retention on the ex vivo porcine tissue.

Prevention of cell adhesion is a major goal of this work, to ensure an efficient barrier is formed between the epicardium and other tissues to properly prevent adhesion formation within the first several weeks post-surgery^4,56^. Following induced trauma, pericardial mesothelial cells detach from the native tissue layer as blood and inflammatory cells enter the affected area. In the weeks that follow, the increased accumulation of fibrin, collagen, and fibroblasts results in strong adhesive connections between the heart and surrounding tissue^4^. PEG is well known to act as an anti-cell adhesion material and therefore we expected minimal cell adhesion on our oxime hydrogels^57–59^. Cytocompatibility with L929 fibroblasts demonstrated the non-cytotoxic nature of Ald–AO–Cat. It was previously reported that Ald–AO oxime gels were cytocompatible, and therefore it was expected that similar results would be observed in this study. DA has been increasingly studied in recent years in biomaterials with adhesive properties, with similar results^39–43,60^ even though H_2_O_2_ is generated during Cat crosslinking^61^. Therefore we did not expect Cat-8PEG to exhibit any cytotoxicity.

Typically, Cat-functionalized biomaterials are designed for tissue and cell adhesion applications^39,42,51^. Conversely, we have successfully demonstrated the use of a Cat-containing polymer to prevent adhesions from forming between the epicardium and adjacent tissues in the chest cavity. We suspect that our incorporation of Cat functionalization (27 wt% Cat-8PEG) in Ald–AO–Cat ensures rapid binding of Cat to primary amines on the epicardial surface, and incorporation into the gel network, leaving limited unreacted Cat available for adhering to other tissue surfaces. To date, we have not seen a case of Cat used in this manner. From our previous unpublished in vivo studies, we determined that Ald–AO would not adequately be retained on the heart to show efficacy as a cardiac anti-adhesion barrier. By adding 27 wt% of Cat functionalization, we expected to see a reduction of adhesion formation in the in vivo rat model. Re-entry into the chest after 2 weeks revealed reduced adhesion formation and intensity for Ald–AO–Cat compared to Ald–AO and untreated groups. The lower adhesion and intensity scores of Ald–AO–Cat at 2 weeks supported our claim that better retention and slower degradation allow for a more efficient cardiac anti-adhesion barrier system. Better efficacy for reducing adhesion formation and intensity was shown for rats treated with Ald–AO–Cat compared to Ald–AO, and therefore only Ald–AO–Cat was studied at 4 weeks compared to untreated controls. At 4 weeks, a complete reduction of adhesion formation was observed for Ald–AO–Cat rats, indicating its efficacy as a cardiac anti-adhesion barrier. Compared to the 2-week study, the overall lower adhesion scores reported at 4 weeks can be explained as a phenomenon commonly observed in patients. Generally, cardiothoracic surgeons report less pericardial adhesion coverage during re-sternotomy with increased waiting time prior to reoperation. As a limitation of the rat model, the rapid healing time of rats may have also contributed to low adhesion coverage observed at 4 weeks. While there is no clinically used standard for preventing post-surgical cardiac adhesions, which is why we chose to use a non-treated control, the lack of comparison to another commercial adhesion barrier like Seprafilm is another limitation of our studies.

Echocardiography 3 days post-abrasion and material application did not show any signs of cardiac dysfunction. In previous reports of using CoSeal^®^ in clinical trials, the 400% swelling caused cardiac tamponade for several patients early in the study^13,14^. Cardiac tamponade is a serious medical condition, resulting in compression on the heart and decreased cardiac function^62^. Our oxime hydrogel was designed with 8-arm star PEG components, to allow for rapid gelation with many crosslinking sites, which ultimately results in minimal swelling and maintenance of cardiac function.

Histological analysis was conducted at 4 weeks post material application to visualize any inflammation occurring on the epicardial surface in response to Ald–AO–Cat. Thin encapsulation occurred around the perimeter of the gel, which is expected for a foreign material implanted in vivo. As expected, we did not see any indication of cell infiltration into the gel, resulting from the anti-protein adsorption properties of PEG^57,58^. High-resolution images of the capsule formation indicated some macrophage presence. This was also observed for CoSeal^®^, another PEG-based polymer, in a porcine cardiac adhesion model^1^. However, no lymphocytes or neutrophils were present with our oxime hydrogel, suggesting that Ald–AO–Cat did not cause chronic inflammation when administered on the epicardium. Given the thin encapsulation and lack of histological evidence of chronic inflammation, this suggests that the Ald–AO–Cat does not impair the normal wound healing process. These findings are consistent with studies that showed the biocompatibility of Cat functionalized materials used as tissue adhesives^63,64^. Histological analysis also revealed that Ald–AO–Cat was not fully degraded up to 4 weeks post-delivery, providing a suitable barrier for reducing or eliminating the formation of cardiac adhesions. Cardiac adhesions are typically formed within the first 30 days after an open-heart procedure. The early and intermediate postoperative phases (1–30 days) are characterized by accumulation of fibrin, inflammatory cells, and fibroblasts at the site of surgical trauma, and depressed fibrinolysis, allowing for the formation of fibrous tissue adhesions between the heart and adjacent tissues^4,65^. Disrupting this process during the first 30 days post-op with a barrier device is an effective way to prevent adhesion formation.

Finally, we developed a spray device that allowed simultaneous mixing and application of the two polymer solutions in a pilot porcine study. Arrangement of all channels for fluid flow in the assembled device ensured that airflow through each nozzle in the upper portion atomizes each liquid when pressure is applied to the syringes attached to the lower piece. Separation of the component solutions within the device is important due to the rapid gelation time of the PEG hydrogel which may impair delivery if mixing occurs prematurely. The overlapping trajectories of the droplets from both pairs of nozzles interact on the heart surface to form the gel layer. Testing of the spraying device indicated that adequate mixing of the two solutions occurred at a working distance of 12–17 cm leading to a homogenous gel formation and epicardial coverage, with minimal overspray, on the ex vivo heart. It is also important to note that with 4 mL of each solution being delivered, a 0.5–1 mm-thick gel was formed, which mimics the application of CoSeal^®^ in pre-clinical porcine models^1^. Furthermore, the gel could be easily removed manually from the tissue surface, which would be important in the case a surgeon needs emergency re-access to the heart.

Compared to the non-treated controls, the pilot porcine study suggests the use of Ald–AO-Cat as a potential option for reducing the severity of cardiac adhesions in a clinical setting, although, given the small pilot nature of the study, the results are not definitive. The cardiac adhesion model that has been utilized here consistently resulted in the formation of thick, fibrous adhesions that were difficult to remove and required sharp dissection. This is comparable to the adhesion model used previously to perform pre-clinical studies with CoSeal^®^, which resulted in nearly identical adhesion scores in the control group^1^. Application of Ald–AO–Cat resulted in a reduction in adhesion severity, with the majority of the adhesions only requiring easy blunt dissection, and no tissue damage when they were removed. Compared to previous preclinical studies with CoSeal^®^, this pilot data suggest improved adhesion severity scores^1^. It should be noted that in pediatric clinical trials, CoSeal^®^ application was related to serious adverse events including cardiac tamponade, vena cava occlusion, and cardiac fibrillation, which were attributed to the large swelling capacity (400% swelling) of CoSeal^[®14^. Here, we have mimicked the 8 mL dosing of CoSeal^®^ used in the pre-clinical porcine study^1,59^, and have shown indications of better reduction of adhesion severity, when scored on similar scoring systems; however, given our study was only a pilot, a larger powered study will be necessary to fully evaluate efficacy in the large animal model. Because of the minimal swelling of Ald–AO–Cat compared to CoSeal^®^ a future dosing study will allow us to determine if delivering larger amounts of oxime hydrogel will result in more favorable results, whereas clinical trials revealed that CoSeal^®^ dosing was largely restricted by the inherent swelling behavior^14^. As mentioned previously for the rat model, adhesion coverage also shows a decreasing trend from 3 to 6 weeks in the porcine model, which is commonly observed in patients. Taken together, the rodent data along with the pilot pig data support the motivation for future dosing and efficacy studies of Ald–AO–Cat for cardiac adhesion prevention and reduction and continued translational development.

In this study, we have provided evidence of a potential solution to the problem of post-surgical cardiac adhesions. When attempting to approach this condition using a physical barrier applied to the heart surface, it is important to consider the inherent properties of a proposed hydrogel in addition to its behavior when used in both in vitro and in vivo environments. We have demonstrated here that the Ald–AO–Cat hydrogel possesses mechanical characteristics and degradation kinetics that are well-suited to the conditions within the chest cavity. The material also demonstrated a lower degree of swelling that surpasses the behavior of existing products. The ability of this biocompatible Ald–AO-Cat hydrogel to prevent adhesion formation is demonstrated by reducing the onset of post-surgical adhesions in vivo. Pilot porcine cardiac anti-adhesion data support further investigation of this hydrogel device for dosing and efficacy for potential future clinical translation.

## Methods

### Materials

Alcohol-terminated 8-arm PEG (molecular weight (MW 10 kDA) was purchased from Creative PEGWorks (North Carolina, USA). Alexa Fluor^®^ 594 NHS Ester (Succinimidyl Ester) and Alexa Fluor^®^ 594 Hydrazide were purchased from Thermo Fisher Scientific. All other chemicals were purchased from Sigma-Aldrich and were used as received without further purification.

### Synthesis of 8-arm aldehyde-PEG (Ald-8PEG)

Synthesis and purification of Ald-8PEG were performed as previously reported^46^. Alcohol-terminated 8-arm PEG (MW 10 kDa) (1.0 g, 0.1 mmol) was dissolved in anhydrous dichloromethane (20 mL) and then 4-carboxybenzaldehyde (360.3 mg, 2.4 mmol) was added. The reaction flask was placed into an ice bath, and then *N*-(3-dimethylaminopropyl)-*N*′-ethylcarbodiimide hydrochloride (460.1 mg, 2.4 mmol) and 4-(dimethylamino)pyridine (13.0 mg, 0.1 mmol) were added, followed by the addition of methanol (0.5 mL) 48 h later. After 3 h of stirring, the reaction solution was dialyzed (molecular weight cut off 3500 g/mol) against methanol to yield Ald-8PEG. ^1^H NMR (Ald8PEG, 300 MHz, CDCl_3_) δ: 10.08 (s, 8H, CHO), 8.19 (m, 16H, C_6_H_4_), 7.93 (m, 16H, C_6_H_4_), 4.49 (m, 16H, CH_2_OCHO), 3.85–3.35 (m, CH_2_CH_2_OCHO and PEG protons). NMR was performed using a 400 Varian Mercury Plus with VNMRJ 4.2 Software.

### Synthesis of 8-arm aminooxy-PEG (AO-8PEG)

Synthesis and purification of AO-8PEG were performed as previously reported^46^. Alcohol-terminated 8-arm PEG (MW 10 kDa) (1.0 g, 0.1 mmol) was dissolved in anhydrous dichloromethane (20 mL) and tetrahydrofuran (2 mL), and *N*-hydroxyphthalimide (407.3 mg, 2.5 mmol) and triphenylphosphine (655.7 mg, 2.5 mmol) were added. After placing the reaction flask into an ice bath diisopropyl azodicarboxylate (427.5 μL, 2.4 mmol) was added dropwise over 30 min, followed by the addition of methanol (0.5 mL) 48 h later. After 3 h of stirring, the reaction solution was dialyzed (molecular weight cut off 3500 g/mol) against methanol to yield 8-arm *N*-hydroxyphthalimide-PEG. *N*-hydroxyphthalimide-PEG (331.0 mg, 0.033 mmol) was then dissolved in acetonitrile (5.296 mL) and hydrazine monohydrate (121.8 μL, 2.5 mmol) was slowly added over 15 min. After 2 h of stirring, dichloromethane (46 mL) was added and filtered over a plug of Celite^®^ 545 to yield AO-PEG. ^1^H NMR (AO8PEG, 300 MHz, D_2_O) δ: 3.88–3.85 (m, 2H, CH_2_ONH_2_), 3.8–3.4 (m, PEG protons). Ald-8PEG and AO-8PEG were stored under vacuum.

### Synthesis of 8-arm Cat-PEG

#### Synthesis of NPC-activated 8-arm PEG (NPC-8PEG)

Alcohol-terminated 8-arm PEG (MW 10 kDa) (5.00 g, 0.50 mmol) was dissolved in anhydrous dichloromethane (100 mL). The reaction flask was placed into an ice bath followed by addition of NPC (8.06 g, 40.00 mmol), trimethylamine (TEA) (5.58 mL, 40.00 mmol) and 4-(dimethylamino)pyridine (DMAP) (0.81 g, 4.00 mmol). After 3 h the reaction solution was poured into cold diethyl ether (900 mL), and the orange precipitate was collected via filtration and washed with cold diethyl ether. NPC-8PEG was obtained as a yellow solid. (5.65 g, 99.81% yield). ^1^H NMR (NPC8PEG, 300 MHz, CDCl_3_, δ): 8.26 (m, 16H, C_6_H_4_), 7.38 (m, 16H, C_6_H_4_), 4.43 (m, 16H, CH_2_OCOO), 3.85–3.35 (m, CH_2_CH_2_OCOO and PEG protons).

#### Synthesis of 8-arm Cat-PEG (Cat-8PEG)

NPC-8PEG (5.65 g, 0.50 mmol) was dissolved in anhydrous 1-methyl-2-pyrrolidone (113 mL), followed by addition of DA hydrochloride (7.57 g, 39.93 mmol) and TEA (5.57 g, 39.93 mmol). The reaction flask was kept at 60 °C for 12 h. The crude reaction product was dialyzed (MW cut off 3500 g/mol) against water (pH = 3–4) to prevent the oxidative polymerization of DA, followed by filtration and lyophilization. Cat-8PEG was obtained as a solid (3.35 g) with 74% functionalization according to ^1^H NMR. ^1^H NMR (Cat8PEG, 300 MHz, DMSO-d_6_) δ: 7.20 (m, 8H, NH), 6.59 (m, 8H, C_6_H_4_), 6.53 (m, 8H, C_6_H_4_), 6.39 (m, 8H, C_6_H_4_), 4.03 (m, 16H, CH_2_OCONH), 3.85–3.35 (m, CH_2_CH_2_OCONH and PEG protons). 3.40–3.05 (m, 32H, NHCH_2_CH_2_ and NHCH_2_CH_2_). Cat-functionalization was confirmed using a colorimetric DOPA assay developed by Waite and Benetict^66^. In brief, a known amount of Cat-8PEG was dissolved in dilute acid. 100 µL of Cat-8PEG solution was added to 300 µL 0.5 M HCl in an Eppendorf tube. 300 µL of nitrite reagent (1.45 M sodium nitrite and 0.41 M sodium molybdate) and 400 µL of 1 M NaOH were added in rapid succession to the tube to form a red solution. Samples were read at 500 nm using a microplate reader (BioTek) within 2–5 min of NaOH addition. A standard curve was generated using known concentrations of 3,4-dihydroxyphenylalanine (*L*-DOPA) (Supplementary Fig. 4).

### Preparation of hydrogels

Hydrogels were prepared in water or 1× PBS, as specified for each experiment. In brief, for oxime hydrogels (Ald–AO), Ald-8PEG and AO-8PEG solutions were prepared and mixed to obtain final hydrogel solutions with a 1:1 ratio of the two polymer. For Cat-oxime hydrogels (Ald–AO–Cat), AO-8PEG solution was prepared separately from the Ald-8PEG and Cat-8PEG (Ald/Cat-8PEG) solution of the desired Cat-8PEG to Ald-8PEG ratio (Table 2). The two solutions were mixed to obtain a gel with the desired ratios of all three polymers. In vivo, each polymer solution was individually loaded into syringes and connected to a gas-assisted dual spray applicator tip (Nordson Medical, Loveland, CO, USA).

### Characterization of hydrogels

Two polymer solutions (200 μL) (AO-8PEG and Ald-8PEG or Ald/Cat-8PEG) were added simultaneously to 4 mL glass vials and the timer started. Gelation time was measured at the inability of the solution to flow using a vial tilting method (*n* = 3 for each formulation)^46,50^. Gelation was measured by hand for these gel formulations. Each hydrogel was incubated at 37 °C for 24 h and then placed into PBS pH 7.4 with 9-fold volume for 24 h. The swelling ratio (*Q*) was calculated as follows: *Q* = *V*_s_*/V*_i_*100 (%), where *V*_s_ is the volume of swollen hydrogels after 24 h and *V*_i_ is the initial volume of hydrogels immediately after gelation. The swelling ratio of each hydrogel reached a plateau within 24 h and was defined as the equilibrium swelling ratio (*n* = 3 for each formulation).

### Rheological studies

The storage modulus (*G*′) and loss modulus (*G*″) data were acquired using AR-G2 (TA Instruments; Rheology Advanced Instrument Control program v5.7.1) with a 20 mm parallel plate geometry operated at 37 °C by a frequency sweep from 10^−2^ to 10^2^ Hz. The hydrogels (500 μL, *φ* = 21 mm) were prepared on the stage of the rheometer and were measured with the 1200 mm gap between the plate and the stage of the rheometer after 15 min of hydrogel incubation. Relative *G*′ studies were conducted at 0, 1 and 3 h post hydrogel gelation. Relative *G*′ was calculated as follows: Relative *G’* = *G*′_*t*_/*G*′_*t*=0_*100 (%), where *G*′_*t*_ is the *G*_*t*_′ at a given time and *G*′_*t=*0_ is the initial *G*′ after 15 min of gel incubation.

### In vitro degradation studies

The hydrogels (200 μL) were prepared in 4 mL glass vials and then placed into PBS pH 7.4 with 9-fold volume. PBS was replaced once a day and the hydrogels were weighed.

### Ex vivo hydrogel retention on cardiac tissues

Ald-8PEG and AO8-PEG were, respectively, conjugated with Alexa Fluor^®^ 594 dye as previously reported^46^. Ald-8PEG (330 mg) was dissolved in 0.1 M PBS (10 mL, pH 4.0), and Alexa Fluor^®^ 594 Hydrazide (330 μL of 1 mg/mL in dimethyl sulfoxide) was added and incubated at room temperature for 1 h. AO-8PEG (300 mg) was dissolved in 0.1 M PBS (10 mL, pH 9.0), and Alexa Fluor^®^ 594 Succinimidyl Ester (330 μL of 1 mg/mL in dimethyl sulfoxide) was added and incubated at room temperature for 1 h. Each respective PEG solution was then dialyzed (molecular weight cut off 3500 g/mol) in ethanol. Biopsies of porcine heart within hours of euthanasia were taken with *φ* = 5 mm punches. Each biopsy was dipped into PBS pH 7.4 to mimic the fluid present during a surgical procedure. The hydrogels were then formed using two pipettes (each 5 μL) resulting in 10 μL on the surface of the tissue. After 15 min, biopsies covered with hydrogels were placed into a 48-well plate with PBS (400 μL) containing 0.625% vol/vol penicillin–streptomycin (Thermo Fisher Scientific) to completely cover the gel-coated tissue. Buffer was immediately removed, and fluorescence was measured on a microplate reader (BioTek) (excite 589 nm, emission 615 nm, sensitivity 100), and fresh buffer was added (*n* = 3 for each formulation). Cat-8PEG was not conjugated with the dye so fluorescence was derived from Ald-8PEG and AO-8PEG in the hydrogels.

### In vitro retention of Cat-8PEG in Ald–AO–Cat gels

Hydrogels were formed in 24-well plates with 100 µL Ald–Cat-8PEG in 1× PBS (200 and 150 mg/mL) and 100 µL AO-8PEG in 1× PBS (200 mg/mL). Gels were incubated at room temperature for 10 min. and were then washed with 1× PBS (500 µL) for 10 min on an orbital shaker at 50 rpm. The supernatant was collected (10 min time point), and a new 1× PBS (500 µL) was added. Samples were incubated at 37 °C for upto 7 days, with 1× PBS changes each day. The supernatant was collected at 10 min, 1, 2, 3, and 7 d post-gelation to assess the release of Cat-8PEG. Day 7 gels were collected at the end of the experiment. Cat-8PEG concentrated in the supernatant and day 7 gels were detected using a colorimetric DOPA assay as previously reported^66^. The supernatant samples were lyophilized and resuspended in 200 µL of dH_2_O. 50 µL of this solution was diluted 1:4 in 1× PBS. 100 µL 0.5 M HCl, 300 µL of nitrite reagent (1.45 M sodium nitrite and 0.41 M sodium molybdate), and 400 µL of 1 M NaOH were added in rapid succession to the Eppendorf tube to form a red solution. Samples were read at 500 nm using a Synergy 4 Multi-mode Microplate Reader (BioTek) within 2–5 min of NaOH addition. The same procedure was used for the day 7 gels (note: digestion of the gels does occur during this method, however complete digestion was not achieved, therefore trapping some Cat-8PEG in the remaining gel and reducing the absorbance reading.) A standard curve was generated using known concentrations of Cat-8PEG (Supplementary Fig. 5A).

### Inflammatory cell adhesion studies

The hydrogels (100 μL) were formed in a 96-well plate. The gels were swollen in Dulbecco’s PBS for 48 h at 37 °C. The membranes of L929 murine fibroblasts and RAW macrophages were labeled with PKH26 (Sigma’s, PKH26 Red Fluorescent Cell Linker Mini Kit for General Cell Membrane Labeling) following manufacturer protocol. The PBS on the swollen gels was removed and either labeled L929 fibroblasts (100 μL, 200,000 cells/cm^2^) or RAW macrophages (100 μL, 100,000 cells/cm^2^) were seeded on top of the gels; TC plastic was used as a control. After 24 h at 37 °C, five fluorescent images were per well. The fluorescence per area was quantified using ImageJ v1.51.

### Direct contact assay

L929 murine fibroblasts were cultured with Dulbecco’s modified eagle’s medium (DMEM) supplemented with 10% fetal bovine serum (FBS) and 0.5% penicillin–streptomycin (P/S) and glutathione (2.61 mg/mL). Cells were allowed to grow to 70–90% confluency before plating. Cells were seeded 16,000 cells/cm^2^ and allowed to reach confluency (~24 h). Once confluent, baseline morphologic and metabolic activity was measured with bright-field images and an alamar blue assay, respectively, for a baseline reading before treatment. The alamar blue assay was used as specified by the manufacturer’s protocol. After baseline measurements were obtained, three treatments were administered to the cells to test cytocompatibility: (1) Ald–AO–Cat gel, (2) positive control, (3) negative control. Ald–AO–Cat gel solutions were prepared as described above and sterilized with a 0.22 µm filter before gelation. Gels were prepared directly on cell layers by combining 17 µL of each solution to the center of the cell layer and allowing 1 min for the material to gel completely and dry. Fresh culture media was added to the wells. Positive and negative controls were prepared by adding zinc diethyldithiocarbamate (ZDEC; 100 mg/ml) and PBS (1:10, v/v) to the new media. After incubation for 24 h at 37 °C and 5% CO_2_, metabolic activity and cell morphology were measured. Micrographs of each sample set were taken for morphological analysis. All groups were blinded prior to grading for healthy cell morphology, based on the criteria for biocompatibility outlined by the International Organization for Standardization (ISO 10993-5). The following grading system was used: Score 0 = No cells appear round in shape or lysed; Score 1 = ≤20% of cells appear rounded with minimal lysing; Score 2 = ≤50% of cells appear rounded with moderate lysing; Score 3 = ≤70% of cells appear rounded with significant lysing; Score 4 = All cells appear rounded and lysed. Following grading, images were unblinded and the scores for each sample group were taken for statistical analysis.

### In vivo rat cardiac anti-adhesion ability of hydrogels

Cardiac anti-adhesion ability was evaluated on male Sprague-Dawley rats (Charles Rivers Laboratories Inc., San Diego, CA, USA) weighing 338 ± 28 g (3–4 months old). All study procedures were approved by the University of California, San Diego Institutional Animal Care and use Committee (Protocol S08172) and performed in an American Association for Accreditation of Laboratory Animal Care (AAALAC) accredited facility (No. 00503). Rats were housed according to standard conditions, were quarantined for at least 48 h after being received, and were provided with food and water ad libitum. All animals were intubated and anesthetized using 5% isoflurane and subsequently shaved around the area of the incision. Subjects were hydrated using a subcutaneous injection of lactated Ringer’s solution (3 mL) before lowering the isoflurane dosage to 2.5%. Animals were treated with 1% lidocaine administered subcutaneously.

Entry into the chest cavity was via thoracotomy. The pericardium was torn upon entry to access the surface of the heart. The tissue was then abraded by poking the exposed surface 100 times with a 30 G syringe needle. This method was chosen for its consistent production in adhesion formation and severity after comparison to other techniques such as rubbing with a cytobrush, scalpel nicks and a decreased number of needle pokes. The heart was then allowed to dry via air exposure for 30 min before delivery of both hydrogel solutions (400 µL total) using a FibriJet gas-assisted applicator head from Nordson Micromedics. Test groups were treated to obtain 400 mL of either Ald–AO or Ald–AO–Cat gel over the tissue while an untreated group was used as a positive control. The gel was then allowed to dry for 10 min prior to closing of the chest cavity. Isoflurane dosage was lowered to 1% following closure of the ribs and turned off completely following final closure. Buprenex (0.05 mg/kg) was injected subcutaneously for pain relief. After 2 or 4 weeks, animals were euthanized via overdose Fatal Plus at 200 mg/kg. At 4 weeks, the hearts were dissected, paraffin embedded, sectioned, and stained with H&E. Samples from each group were blinded and graded by a histopathologist to assess the presence chronic inflammation.

### Dual-delivery device design and testing

A specialized device was developed to deliver each component together in a 1:1 ratio in the form of an atomized spray produced using compressed air and attached nozzle heads. This working prototype has two separate parts, one of which allows the flow of air through two separate pathways 0.4 cm in diameter after attachment to a compressor at an inlet while a second piece supports the addition of two 10 mL syringes containing sterile PBS solutions of the component polymers injected into the path of air through two pathways with a diameter of 0.2 cm. All nozzle heads have a diameter of 0.05 cm. The device was designed in SolidWorks and made with PLA filament on a Prusa i3 MK2 3D printer. The device was sterilized using EtO 48 h before the procedure. For in vivo use, the device was operated at a gauge pressure of 10 psi at ~10–12 cm from the target surface while gel formation was visualized by adding India ink to each polymer solution (1:100 v:v).

The mixing of two solutions at a 1:1 ratio was confirmed with absorbance spectroscopy using dyed aqueous sugar solutions to mimic the viscosities of the AO-8PEG (0.01 Pa s) and Ald–Cat-8PEG (0.04 Pa s) solutions. Yellow dye with a peak absorbance at 420 nm was added to the PEG-AO substitute solution while a blue dye with a maximum at 610 nm was added to the PEG-Ald/PEG-DA substitute (2:25, v-v). A standard solution made from 250 µL of each dyed substitute was used as a control. The device was tested at working distances of 10, 12.5, 15, and 20 cm by spraying both solutions simultaneously into a Petri dish 5.5 cm in diameter and collecting 500 µL of the final mixed solution. Absorbance spectroscopy performed using a TECAN plate reader measured the signal of each sprayed mixture at both 420 and 610 nm.

### In vivo porcine cardiac adhesion pilot study

Cardiac anti-adhesion ability was evaluated on male KG Farm Pig (S & S Farms, Ramona, California) weighing 15–27 kg (~1 month old) in a small pilot study. All study procedures were approved by the University of California, San Diego Institutional Animal Care and use Committee (Protocol S09168) and performed in an American Association for Accreditation of Laboratory Animal Care (AAALAC) accredited facility (No. 00503). Pigs were housed according to standard conditions, quarantined for at least 72 h after being received, and were provided with food and water ad libitum. A Fentanyl patch (50 mcg) was placed for pain control 24 h prior to median sternotomy. A combination of ketamine (20–30 mg/kg)/xylazine (2 mg/kg)/atropine (0.04–0.05 mg/kg) was administered IM for pre-anesthesia induction. All animals were intubated and anesthetized with Propofol (2.4 mg/kg) with 1–3% isofluorane administration maintained throughout the surgical procedure for deep anesthesia.

A median sternotomy (up to 20 cm incision) was performed to gain entry into the chest cavity. The pericardial sac was cut open and the heart was fully exposed. Adhesions induction was conducted by rubbing gauze 15–20× on five regions of the anterior surface of the heart. 5 mL of blood was removed from the right atrium and sprayed on the epicardium and the chest cavity was left open to dry, exposed to air, for 15 min. Ald-Cat and AO solution in 1× PBS were prepared fresh and in a sterile hood prior to spraying. A total of 4 mL of each solution with India Ink (1:100) were prepared, filtered with a 0.22 µm sterile filter, and loaded into the spraying device. Fluid pooled in the chest cavity was aspirated prior to administering Ald–AO–Cat gel. Ald–AO–Cat gel (8 mL total) was sprayed onto the heart surface creating a 0.5–1 mm-thick gel (working pressure of 10 psi and a working distance of ~12–17 cm). After spraying, the chest was left open, exposed to air for 10 min before closing. Control animals received the same treatment, without the spray of a gel. The pericardium was left open, sternal wires were used to close the chest, and sutures were used to for closing subcutaneous and skin layers. Fentanyl (10 mcg/kgm, IV), Baytril (10 mg/kg, IM), and Flunixin (2 mg/kg, IM) was administered intra-operatively and Bupivicain was injected at the incision site before wrapping the chest with gauze. Fentanyl (5 mg/kg) was delivered IV during recovery. Carprofen (2 mg/kg, IM, SID) for 3 days post-op and Clavamox (375 mg, PO BID) was delivered for 5–7 days post-op.

Pigs were euthanized at 3 and 6 weeks post sternotomy with an overdose of Fatal-Plus (9 mL). Pigs that received Ald–AO–Cat spray were euthanized at 3 (*n* = 1) and 6 (*n* = 1) weeks post sternotomy. The control pigs (*n* = 3) were euthanized 6 weeks post sternotomy. Each animal was euthanized followed by resternotomy and adhesion gross assessment. Images of the anterior hearts surface immediately following resternotomy were used for blinded adhesion grading of coverage. Adhesion strength was assessed during resternotomy. Images from resternotomy were segmented into six equivalent sections (similar to the rat grading criteria), and each section was graded on a scale of 1–4 for adhesion coverage and strength as shown in Table 3. The scoring system was adapted from several surgical adhesion studies^47–49,59,67,68^.

### Statistical analysis

Statistical differences between the experimental data for the 4 weeks in vivo adhesion assessment were analyzed using a Mann–Whitney *t*-test. All rheological data, cell adhesion, and cytocompatibility, 2 weeks in vivo data were analyzed using one or two-way analysis of variance (ANOVA) as indicated with Tukey’s post hoc analysis. Statistical significance was set to *p* < 0.05. Results are presented as mean ± standard deviation.

### Reporting summary

Further information on research design is available in the Nature Research Reporting Summary linked to this article.

## Supplementary information

Supplementary Info

Reporting Summary

## Data Availability

All data generated for this study are included in the main and supplementary figures. Other data that support the findings of this study are available on reasonable request from the corresponding author. Source data are provided with this paper.

## Source data

Source Data
