# Supplementary material for: Preventing post-surgical cardiac adhesions with a catechol-functionalized oxime hydrogel
Source: Nat Commun. 2021 Jun 18;12:3764. doi: 10.1038/s41467-021-24104-w (PMC8213776; doi:10.1038/s41467-021-24104-w)
Supplement: Supplementary file 1 — Supplementary Info [file 41467_2021_24104_MOESM1_ESM.pdf]

Supplementary Information

Preventing post-surgical cardiac adhesions with a catechol-functionalized oxime hydrogel

Masaki Fujita, Gina M. Policastro, Austin Burdick, Hillary T. Lam, Jessica L. Ungerleider, Rebecca L. Braden, Diane Huang, Kent G. Osborn, Jeffrey H. Omens, Michael M. Madani and Karen L. Christman

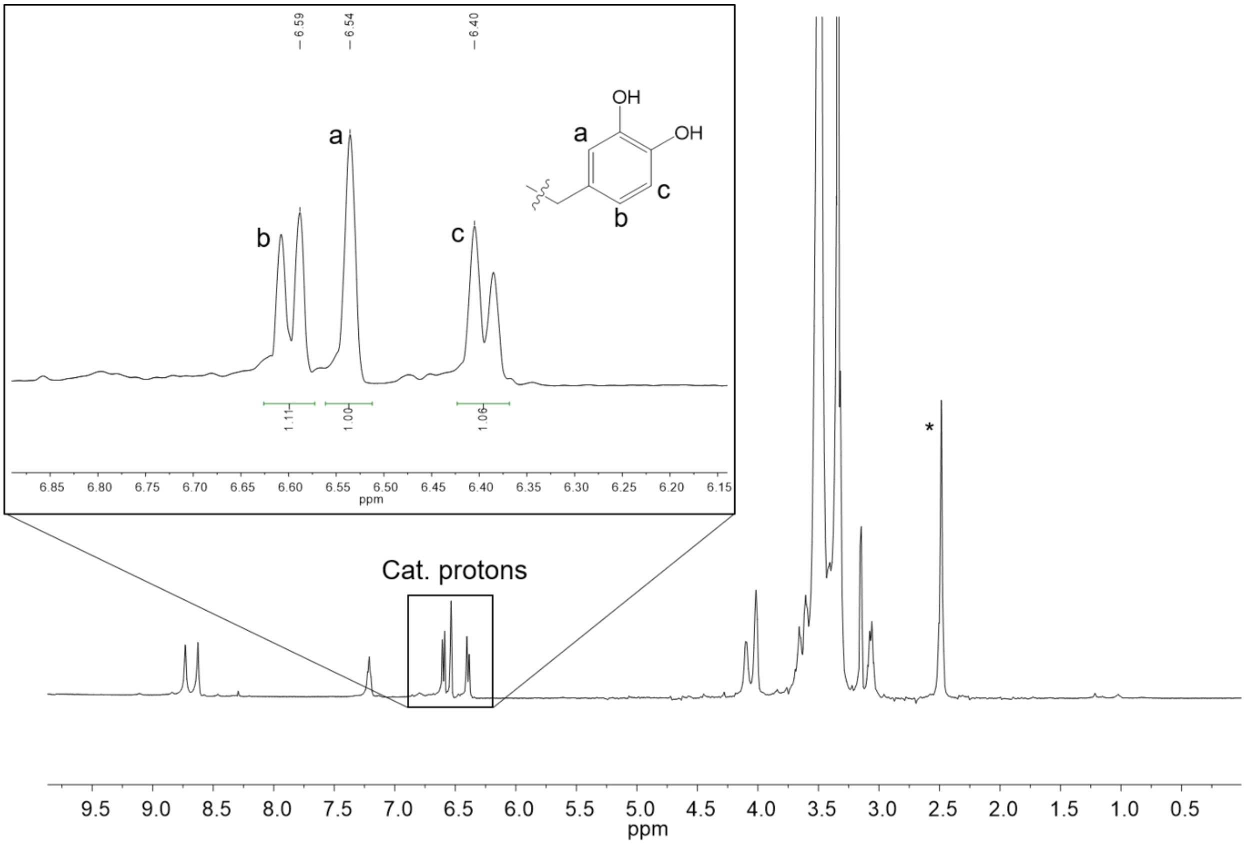

Supplementary Figure 1. Characterization of Cat-8PEG. ^{1}H NMR was used to determine the successful synthesis of Cat-8PEG and determine the functionality ratio. Characteristic peaks at δ= 6.59 ppm, 6.54 ppm and 6.40 ppm are representative of the DOPA catechol group functionalization. Cat8PEG was obtained as a solid (3.35 g) with 74 % functionalization. ^{1}H NMR (Cat8PEG, 300 MHz, DMSO-d_6 (*)) δ: 8.73 (s, 8H, O*H*), 8.63 (s, 8H, O*H*), 7.20 (m, 8H, N*H*), 6.59 (m, 8H, C_6H_4), 6.53 (m, 8H, C_6H_4), 6.39 (m, 8H, C_6H_4), 4.10-4.02 (m, 32H, CH_2CH_2OCONH), 3.85-3.35 (m, PEG protons). 3.15-3.05 (m, 32H, NHCH_2CH_2). Cat-8PEG was stored under vacuum.

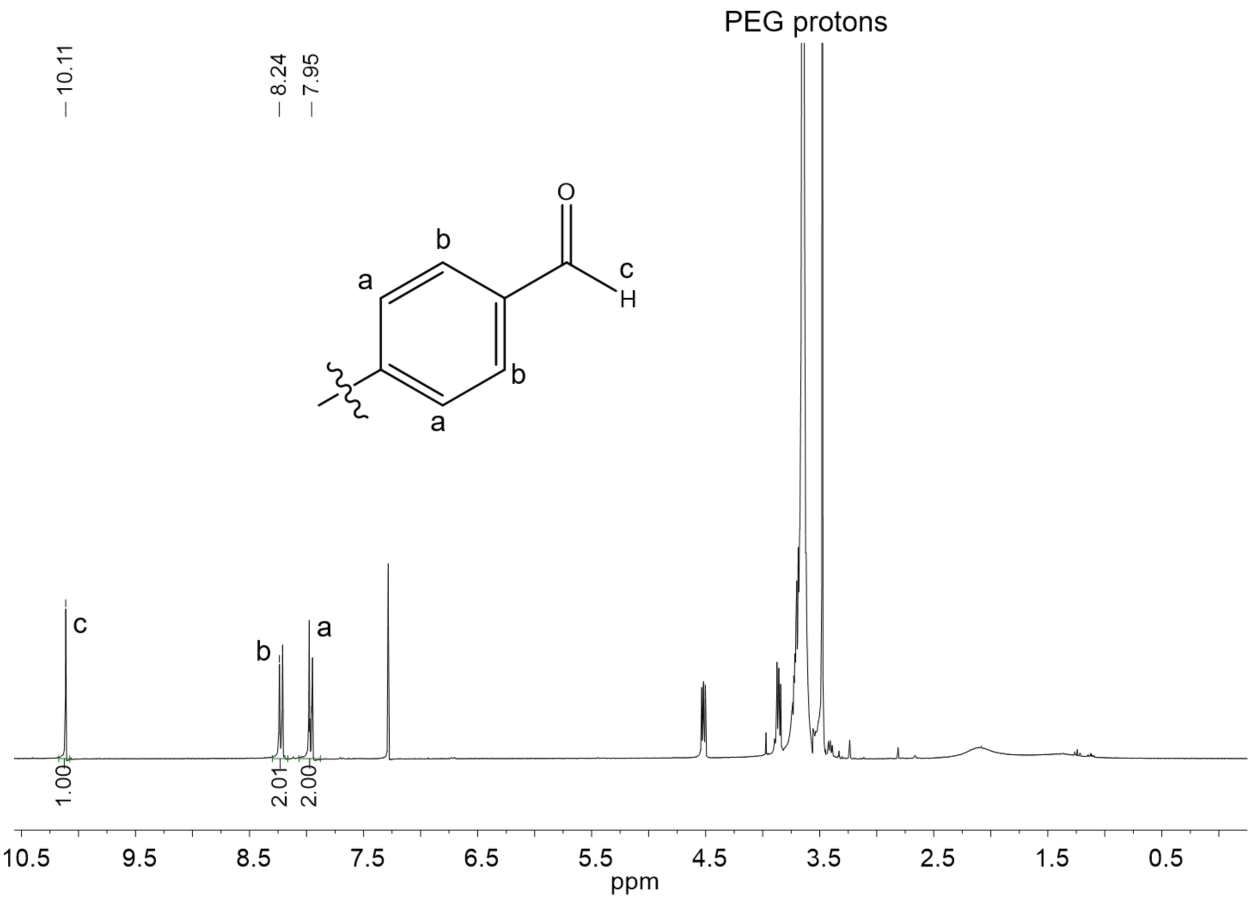

Supplementary Figure 2. Characterization of Ald-8PEG. ^{1}H NMR was used to determine the successful synthesis of Ald-8PEG and determine the functionality ratio. Characteristic peaks at δ= 10.11 ppm, 8.24 ppm and 7.95 ppm are representative of the benzaldehyde functionality. Ald-8PEG was obtained as a white solid with 97 % functionalization. ^{1}H NMR (Ald-8PEG, 300 MHz, $CDCl_3$) δ: 10.11 (s, 8H, C*H*O), 8.24 (m, 16H, C_6H_4), 7.95 (m, 16H, C_6H_4), 4.49 (m, 16H, CH_2OCHO), 3.85-3.35 (m, CH_2CH_2OCHO and PEG protons). Ald-8PEG was stored under vacuum until use.

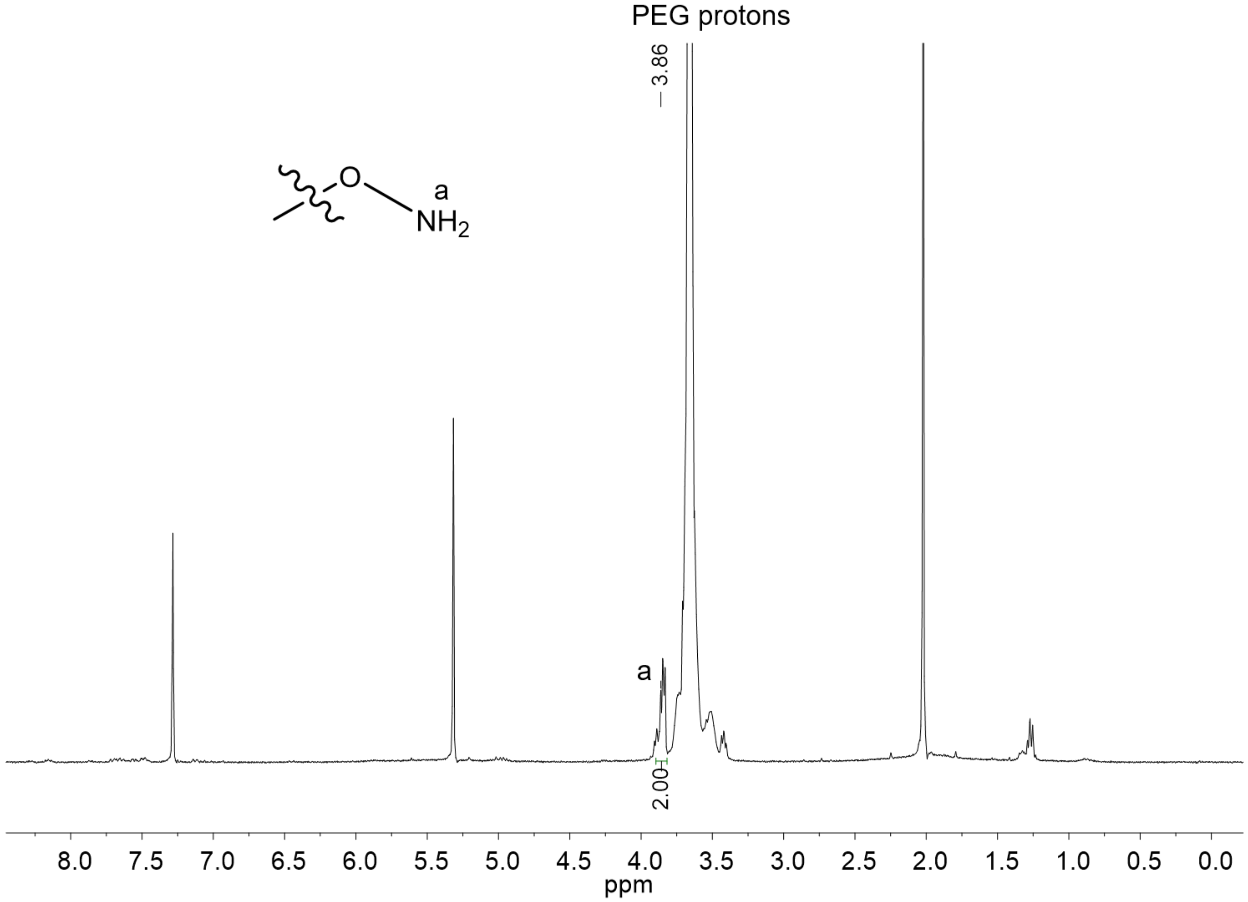

Supplementary Figure 3. Characterization of AO-8PEG. ^{1}H NMR was used to determine the successful synthesis of AO-8PEG and determine the functionality ratio. The characteristic peak at δ= 3.86 ppm represents the aminooxy functionality. AO-8PEG was obtained as a white solid with 83 % functionalization. ^{1}H NMR (AO-8PEG, 300 MHz, D_2O) δ: 3.86 (m, 2H, CH2ONH2), 3.8-3.4 (m, PEG protons). AO-8PEG was stored under vacuum until use.

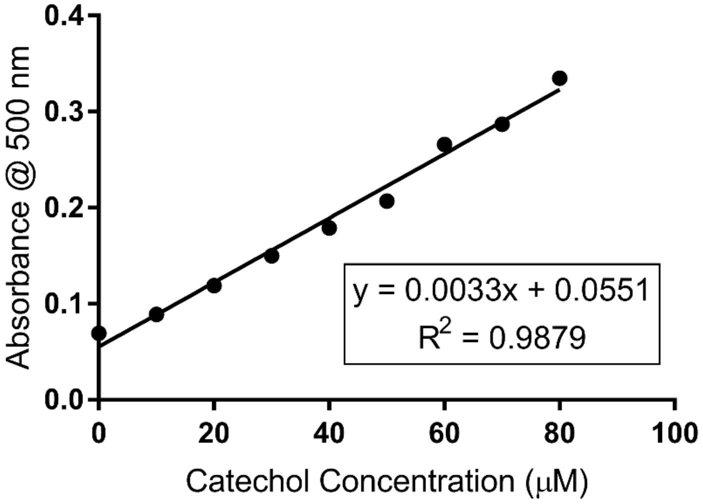

Supplementary Figure 4. Standard curve for quantifying catechol functionalization. A standard curve was generated using known concentrations of 3,4-dihydroxy-L-phenylalanine for quantifying catechol functionalization in Cat-8PEG. Absorbance was recorded at 500 nm. Source data are provided as a Source Data file.

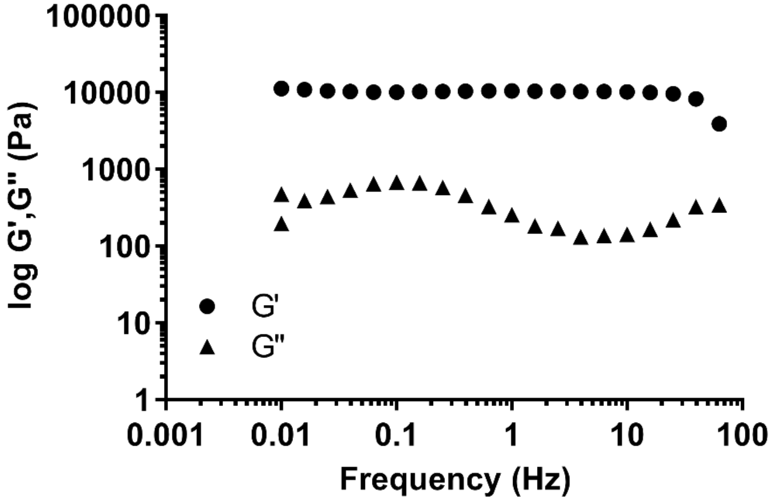

Supplementary Figure 5. Representative *G'* and *G"* across a frequency sweep. This graph is representative of the viscoelastic behavior of both the Ald-AO and Ald-AO-Cat gels which crosslinked and gelled within seconds of combining the two solutions prior to rheometry. *G'* = storage modulus. *G"* = loss modulus. Source data are provided as a Source Data file.

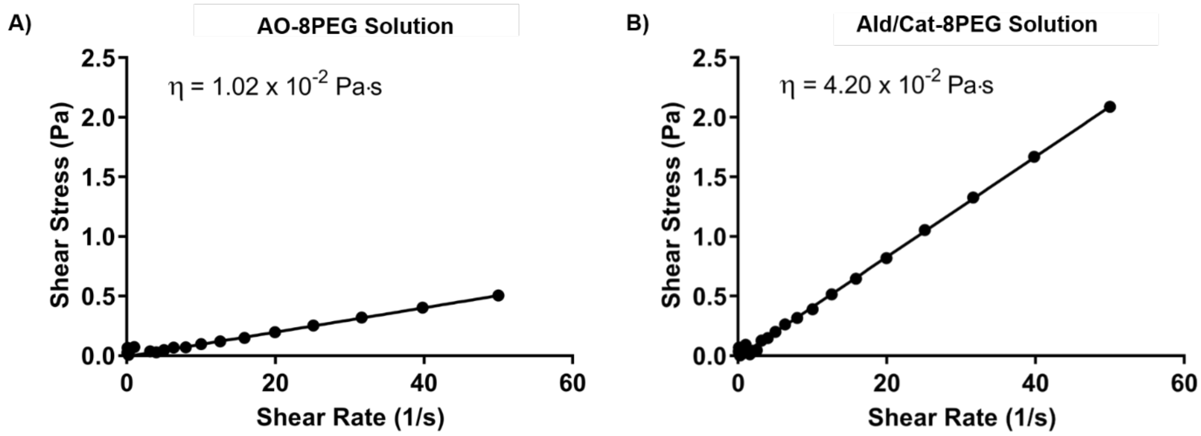

Supplementary Figure 6. Complex Viscosity. Parallel plate rheometry was used to measure the complex viscosity (η) of the Ald-8PEG/Cat-8PEG (A) and AO-PEG (B) solutions. Source data are provided as a Source Data file.

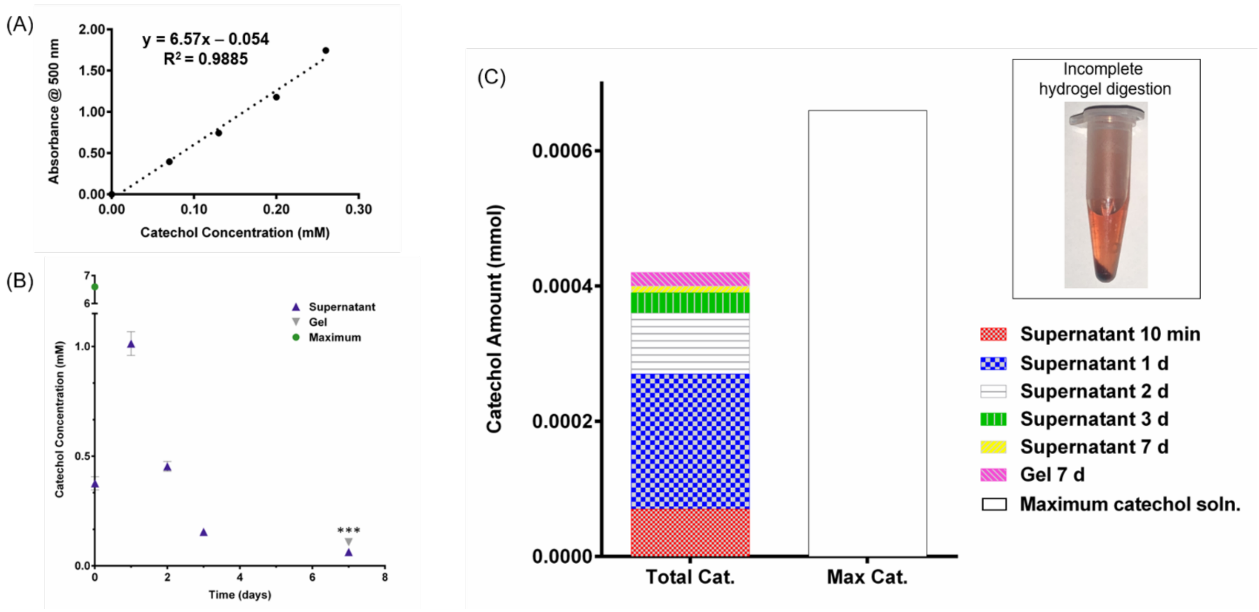

Supplementary Figure 7. Retained catechol in the Ald-AO-Cat as indicated by the time-dependent DOPA-nitration assay. (A) Release of Cat-8PEG was detected in gel supernatant at 10 min, 1 day, 2 days, 3 days, and 7 days post-gelation. An initial release of Cat-8PEG was observed at 10 min, corresponding to the release of Ald-8PEG and AO-8PEG observed in the ex vivo retention assay (Figure 2F). This is indicative of the release of unreacted or non-physically trapped materials. A burst release of Cat-8PEG was detected, similar to the release of Ald-8PEG and AO-8PEG, which was expected with maximum swelling of the oxime hydrogel in aqueous conditions. A time-dependent, decrease in Cat-8PEG release was calculated up to day 7, with a significantly larger amount of Cat-8PEG physically trapped in the gel at 7 days post gelation. (B) The summation of Cat-8PEG released in the supernatant and retained in the gel at day 7, was compared to the total Cat-8PEG concentration in the original gel formulation, with 63% of the total Cat-8PEG accounted for. (C) The remaining Cat-8PEG can be explained by the incomplete digestion of the gels at day 7 resulting in incomplete release of Cat-8PEG for detection. Source data are provided as a Source Data file.

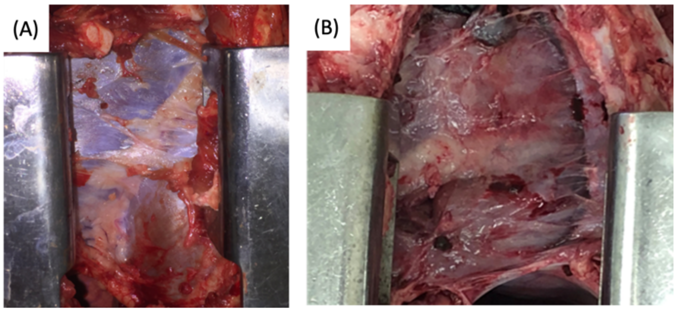

Supplementary Figure 8. Representative images from pilot pig study showing robust adhesions in the control group. Adhesions in the control group required sharp dissection (A), whereas Ald-AO-Cat treated pigs (B) had milder adhesions that required only manual/blunt dissection. B displays Ald-AO-Cat treated pig euthanized at 3 weeks after application.
