# Supplementary material for: Preventing post-surgical cardiac adhesions with a catechol-functionalized oxime hydrogel
Source: Nat Commun. 2021 Jun 18;12:3764. doi: 10.1038/s41467-021-24104-w (PMC8213776; doi:10.1038/s41467-021-24104-w)
Supplement: Supplementary file 2 — Reporting Summary [file 41467_2021_24104_MOESM2_ESM.pdf]

natureresearch

Corresponding author(s): Karen L. Christman

Reporting Summary

Nature Research wishes to improve the reproducibility of the work that we publish. This form provides structure for consistency and transparency in reporting. For further information on Nature Research policies, see Authors & Referees and the Editorial Policy Checklist.

Statistical parameters

When statistical analyses are reported, confirm that the following items are present in the relevant location (e.g. figure legend, table legend, main text, or Methods section).

n/a	Confirmed	
	x	The exact sample size (n) for each experimental group/condition, given as a discrete number and unit of measurement
x		An indication of whether measurements were taken from distinct samples or whether the same sample was measured repeatedly
	x	The statistical test(s) used AND whether they are one- or two-sided<br>*Only common tests should be described solely by name; describe more complex techniques in the Methods section.*
x		A description of all covariates tested
x		A description of any assumptions or corrections, such as tests of normality and adjustment for multiple comparisons
	x	A full description of the statistics including central tendency (e.g. means) or other basic estimates (e.g. regression coefficient) AND variation (e.g. standard deviation) or associated estimates of uncertainty (e.g. confidence intervals)
	x	For null hypothesis testing, the test statistic (e.g. F, t, r) with confidence intervals, effect sizes, degrees of freedom and P value noted<br>*Give P values as exact values whenever suitable.*
x		For Bayesian analysis, information on the choice of priors and Markov chain Monte Carlo settings
x		For hierarchical and complex designs, identification of the appropriate level for tests and full reporting of outcomes
x		Estimates of effect sizes (e.g. Cohen's d, Pearson's r), indicating how they were calculated
	x	Clearly defined error bars<br>*State explicitly what error bars represent (e.g. SD, SE, CI)*

Our web collection on statistics for biologists may be useful.

Software and code

Policy information about availability of computer code

Data collection	Rheological Studies - AR-G2 rheometer (TA Instruments), Rheology Advanced Instrument Control program v5.7.1<br>NMR- 400 Varian Mercury Plus, VNMRJ 4.2 Software<br>In vitro Retention and Alamar Blue assay- Microplate reader (BioTek), Gen5 Software
Data analysis	GraphPad Prism 7, Image J v1.51

For manuscripts utilizing custom algorithms or software that are central to the research but not yet described in published literature, software must be made available to editors/reviewers upon request. We strongly encourage code deposition in a community repository (e.g. GitHub). See the Nature Research guidelines for submitting code & software for further information.

Data

Policy information about availability of data

All manuscripts must include a data availability statement. This statement should provide the following information, where applicable:

- Accession codes, unique identifiers, or web links for publicly available datasets
- A list of figures that have associated raw data
- A description of any restrictions on data availability

The data that support the findings of this study are available from the corresponding author upon reasonable request.

Field-specific reporting

Please select the best fit for your research. If you are not sure, read the appropriate sections before making your selection.

☒ Life sciences ☐ Behavioural & social sciences

For a reference copy of the document with all sections, see nature.com/authors/policies/ReportingSummary-flat.pdf

Life sciences

Study design

All studies must disclose on these points even when the disclosure is negative.

Sample size	Sample size was determined using a power analysis using the strictest power (95%) and $p < 0.05$
Data exclusions	No data points were excluded.
Replication	Studies were performed with appropriate replicates and in vivo studies used power analysis to determine necessary n. Two in vivo rat studies were performed, both showing reduction of adhesions. All studies and replicates are shown.
Randomization	Animals were randomly selected to recieve the various treatments. There was no predetermination of treatment.
Blinding	Investigators were blinded during adhesion scoring and data analysis.

Materials & experimental systems

Policy information about availability of materials

n/a	Involved in the study
☐	☒ Unique materials
☒	☐ Antibodies
☐	☒ Eukaryotic cell lines
☐	☒ Research animals
☒	☐ Human research participants

Unique materials

Obtaining unique materials	Synthesis of the unique, functionalized 8-arm PEG compounds are described in the text. Characterization of these materials is also included.

Eukaryotic cell lines

Policy information about cell lines

Cell line source(s)	ATCC: L929 and RAW
Authentication	Cells lines were not authenticated beyond ATCC
Mycoplasma contamination	Cells were not tested for mycoplasma contamination.
Commonly misidentified lines (See ICLAC register)	None used.

Research animals

Policy information about studies involving animals; ARRIVE guidelines recommended for reporting animal research

Animals/animal-derived materials	Male Sprague-Dawley rats, 3-4 months old; male KG Farm Pig, ~1 month old

Method-specific reporting

n/a	Involved in the study
☒	☐ ChIP-seq
☒	☐ Flow cytometry
☒	☐ Magnetic resonance imaging
